# Pathway-Based Analysis of Genome-Wide siRNA Screens Reveals the Regulatory Landscape of App Processing

**DOI:** 10.1371/journal.pone.0115369

**Published:** 2015-02-27

**Authors:** Luiz Miguel Camargo, Xiaohua Douglas Zhang, Patrick Loerch, Ramon Miguel Caceres, Shane D. Marine, Paolo Uva, Marc Ferrer, Emanuele de Rinaldis, David J. Stone, John Majercak, William J. Ray, Chen Yi-An, Mark S. Shearman, Kenji Mizuguchi

**Affiliations:** 1 Merck Research Laboratories, Merck & Co, Boston, United States of America; 2 Merck Research Laboratories, Merck & Co, Beijing, China; 3 Merck Research Laboratories, Merck & Co, North Wales, United States of America; 4 Merck Research Laboratories, Instituto di Recerca di Biologia Molecolare, Pomezia, Italy; 5 Merck Research Laboratories, Merck & Co, West Point, United States of America; 6 National Institute of Biomedical Innovation, Osaka, Japan; National Center for Geriatrics and Gerontology, JAPAN

## Abstract

The progressive aggregation of Amyloid-β (Aβ) in the brain is a major trait of Alzheimer's Disease (AD). Aβ is produced as a result of proteolytic processing of the β-amyloid precursor protein (APP). Processing of APP is mediated by multiple enzymes, resulting in the production of distinct peptide products: the non-amyloidogenic peptide sAPPα and the amyloidogenic peptides sAPPβ, Aβ40, and Aβ42. Using a pathway-based approach, we analyzed a large-scale siRNA screen that measured the production of different APP proteolytic products. Our analysis identified many of the biological processes/pathways that are known to regulate APP processing and have been implicated in AD pathogenesis, as well as revealing novel regulatory mechanisms. Furthermore, we also demonstrate that some of these processes differentially regulate APP processing, with some mechanisms favouring production of certain peptide species over others. For example, synaptic transmission having a bias towards regulating Aβ40 production over Aβ42 as well as processes involved in insulin and pancreatic biology having a bias for sAPPβ production over sAPPα. In addition, some of the pathways identified as regulators of APP processing contain genes (CLU, BIN1, CR1, PICALM, TREM2, SORL1, MEF2C, DSG2, EPH1A) recently implicated with AD through genome wide association studies (GWAS) and associated meta-analysis. In addition, we provide supporting evidence and a deeper mechanistic understanding of the role of diabetes in AD. The identification of these processes/pathways, their differential impact on APP processing, and their relationships to each other, provide a comprehensive systems biology view of the “regulatory landscape” of APP.

## Introduction

Progressive aggregation of a toxic 42 amino-acid peptide species, known as amyloid-beta (Aβ) 42 is a major hallmark of Alzheimer’s disease (AD). Accumulation of Aβ42 oligomers is thought to cause neuronal injury, synaptic dysfunction, and neuronal death leading to dementia[1,2]. Aβ42 is produced as a result of proteolytic processing of the amyloid precursor protein (APP), a ~100-KDa type I transmembrane protein that is ubiquitously expressed and localized to the trans-Golgi network, endocytic compartments, and cell surface. Sequential cleavage of APP by β- and γ-secretases results in the production of Aβ peptides (Aβ40 and Aβ42) and, hence, several drug-discovery efforts are aimed at finding either β-secretase (BACE1) or γ-secretase inhibitors (GSIs)[3,4]. However, the development of small molecules for either of these targets has proven to be challenging. BACE1 is an aspartyl protease with a large active site that complicates the identification of suitable brain-penetrant small molecules[3,4]. Conversely, several highly potent and cell-permeable GSIs have been identified. However, the development of GSIs has been plagued by mechanism-based toxicities. In addition to APP, the γ-secretase complex has many (>40) other substrates[5], most notably Notch. Proteolytic processing of Notch by γ-secretase results in the release of the Notch intracellular signalling domain (NICD), a process that is inhibited by treatment with GSIs. Chronic inhibition of Notch processing is thought to result in gastrointestinal as well as other toxicities, which are dose limiting in the clinic[3,4,6,7]. Therefore, given the challenges with current therapeutic strategies and the recent identification of protective mutations in APP that lower amyloid levels[8], the identification of alternative ways of regulating Aβ42 production is needed.

Several observations support the notion that alternative approaches to direct BACE1 and γ-secretase inhibition are possible. Oxidative stress, transient interactions with the γ-secretase complex, neuronal activity, cholesterol metabolism, and inflammatory cytokines can directly modulate γ-secretase activity[9]. Therapeutic strategies exploiting many of these processes have been proposed, with some progressing through clinical trials[3,7]. However, the underlying mechanisms by which some of these processes affect Aβ levels are still poorly understood[1,9], complicating the identification and development of novel therapeutic strategies.

Whole-genome siRNA screens provide a powerful means for target identification. By knocking down a gene with siRNA nucleotide probes, one is able to measure the effect on different biological outcomes for thousands of genes simultaneously, enabling the identification of pathways and processes that regulate the biology of interest. However, there are many challenges facing the analysis of siRNA screens. The fact that siRNA probes may not be specific, due to sequence similarity with other genes, results in off-target activities and thus potentially contributes to the high rate of false-positive hits and the low level of reproducibility[10,11]. False negatives are likely to occur due to variations in: (1) probe efficacy; (2) protein stability; and (3) the magnitude of a true biological response, since the position or role of a given gene/protein in a pathway would allow for only a small, yet significant effect on the biology being measured[11–14]. Currently, target (hit) selection is generally handled on an individual basis devoid of any biological context with the main focus on identifying highly active hits defined either by a rank, such as percent activity, or by the use of a Z-score[11,12]. This type of analysis focuses mainly on the extreme values of the distribution representing ~1% of the measured values whilst ignoring the remainder of the data[12]. This led to the development of methods that consider the collective effects of siRNA probes, targeting the same gene, in the identification of likely gene candidates [11,15,16]. Similarly, given that genes belonging to the same pathway/process will act in concert to exert their effect on a biological outcome and that not all genes in a given pathway/process will have uniform effects on biological endpoints, we can leverage pathway or gene set scoring as a complementary approach to the analysis of siRNA screens. Our assumption is that the aggregate effect of genes in a pathway should not occur merely by chance and would also take into account small, but real effects to be considered [17–19].

Here we apply a pathway scoring method to a large-scale siRNA screen aimed at identifying regulators of APP processing. The screen measures the production of APP proteolytic products, the non-amyloidogenic peptide (sAPPα) and the amyloidogenic peptides (sAPPβ, Aβ40, and Aβ42), as well as cell viability[20]. By applying such a method, we were able to derive, for the first time, the “regulatory landscape of APP processing”, identifying most pathways/processes that have been previously implicated in the regulation of APP endoproteolysis, including processes that contain key genetic risk factors identified in recent Genome-Wide Association (GWA) studies for AD[21–25], as well as novel regulatory mechanisms. In addition, we demonstrate how pathway-based analysis can be used to: (1) identify the interplay across different regulatory pathways/processes; (2) understand how production of APP peptides can be regulated by common or distinct mechanisms; (3) propose mechanistic hypotheses as to how a diabetes-related pathway can affect APP processing and (4) provide a framework from which novel amyloid therapeutic strategies could be derived.

## Results

### Identification of pathways that differentially regulate APP processing and cell viability

In order to identify the pathways/biological processes that regulate APP processing and viability, both net and absolute pathway impact (PI) scores were calculated (see Materials & Methods). Briefly, the PI score represents a normalized aggregate effect of all the genes in a given pathway/process. Since genes in a given pathway can either increase or decrease the readout in question by being either “activators” or “inhibitors”, both Net and absolute PI (ABS PI) scores were calculated. The ABS PI score accounts for the scenario where small Net PI scores are obtained due to equal, but opposing effects of genes in a given pathway/process and reflects whether a pathway/process regulates the biological readout independent of the direction of the effect. Net PI and ABS PI scores for pathway/process sets from several public and commercial databases across all biological endpoints measured were calculated (6154 sets in total) (S1–S3 Supplementary Information). Because the PI score may depend on the size of the pathway/process, we also calculated the probability of obtaining such a score by chance for each given set size (see Materials & Methods).

A pathway was considered significant at a *P* ≤ 0.01 for either Net or ABS PI. Table 1 lists the number of pathways/processes identified as significant for each readout (viability, Aβ40, Aβ42, sAPPα, and sAPPβ), respectively. In total, 372 pathway/process sets were identified as significant for at least one of the readouts. Given the central role of Aβ42 in AD pathology, we will focus most of the Results section on pathways identified for this readout and will discuss, when relevant, the results for the other readouts (see Table 1 and S4 and S5 Supplementary Information).

**Table 1:** APP processing landscape.

Readout	Significant Sets (P ≤ 0.01)	Total Number of Genes	Summary of Key Pathways/Process (Table 1)
Viability	111(90)	3192	**1)** Activin A signaling, **TGF-b receptor signaling pathway (CLU)**, receptor protein serine/threonine kinase signaling, senescence, nuclear import, peroxisome transport, IL12 signaling (via STAT4), Immune response (MHC class II), TCR signaling, Osteopontin-mediated events, VEGF signaling, PECAM1 interactions, Macropinocytosis, S1P3 pathway, Integrin signaling (via Grb2:SOS, MAPK; amb2), Blood coagulation, cytoskeleton remodeling, NGF receptor signaling, G-protein signaling (via Rap1A), EphB receptors in dendritic spines, cytoskeleton remodeling, folic acid transport, mitochondrion organization **2)** proteosomal degradation (ubiquitin dependent) **3)** mRNA metabolism (transcription, translation, splicing), Ribosome, Influenze viral replication, glucose homeostasis **4)** copper ion transport, aerobic respiration, response to ATP **5)** Mismatch repair, somatic hypermutation of immunoglobulin genes **6)** Tyrosine metabolism **7)** response to acetate **8)** regulation of angiotensin metabolic process **9)** chitin metabolic process
Aβ40	95(67)	2837	**1)Axogenesis (SORL1); neuron and neurite development (SORL1)**; cell-cell junction maintenance; cell morphogenesis and projection (SORL1); integrin-mediated signaling (via p130Cas and MAPK); autophagy; **PECAM1 interactions (INPP5D)**; memory; Plug formation **2)** nitric oxide metabolism; granulocyte macrophage colony stimulation; **Immunity and defense (CR1);** Natural killer cell mediated immunity, protein destabilization, mRNA metabolism (transcription) **3)**DNA replication (excision, incision) **4)** mRNA metabolism (**transcription (MEF2C)**, translation, splicing), Viral replication **5)** PAK-2p34 proteosome mediated degradation; *Alzheimer's disease pathway*; NRIF cell death signaling **6)** calcium-dependent cell adhesion; **7) synaptic transmission (BIN1)**; detection of chemical stimulus; protein amino acid prenylation **8)** metal transport (Cu^+2^), aerobic respiration, acetyl-CoA metabolism **9)** amino sugar metabolism (N-acetylglucosamine); **10)** selenoamino acid metabolism; peptidyl-arginine methylation
Aβ42	119(82)	2313	**1)** Copper ion homeostatsis, aerobic respiration, metal ion transport **2)** Protein trafficking **3) Immunity and defense (CR1); acute inflammatory response (CR1, CLU);** BMP-signaling pathway; cell adhesion, cell-cell junction organization, nervous system development, plug formation, integrin-mediated signaling (via MPK and Grb2:SOS), post-translational gene silencing. **4)** *ectodomain proteolysis*, *Notch processing*, *Alzheimer's disease pathway*, *Preselinin action in Notch and Wnt signaling*, learning or memory, apoptosis (DSG2) **5)** Neuron recognition, calcium dependent cell adhesion, axonal fasciculation **6)** mRNA metabolism (transcription, translation, splicing), influenza life cycle, and HIV elongation **7)** Selenoamino acid metabolism **8)** Arginine, proline, and triacylglycerol metabolism **9)** Vamp 2,7, 8 associated clathrin vesicle budding **10)** DNA packaging, chromatin assembly, and DNA packaging
sAPPα	154(109)	3084	**1)**;erythrocyte development; EGFR signaling; steroid hormone receptor signaling; Notch receptor processing and signaling; ectodomain proteolysis;amyloid precursor protein processing; Rho GTPase regulation; signaling by BMP; B-cell mediated immunity; T-helper 2 cell differentiation; Interferon-gamma production; Ras signaling in CD4+TCR pathway; **cartilage development (MEF2C)**; osteoclast differentiation; **myeloid leukocyte differentiation (TREM2); chondrocyte differentiation(MEF2C)**; regulation of transcription; **positive regulation of cell proliferation (PTK2B, SORL1) 2)** heart contraction; phosphatidic acid metabolism; role of ZNF202 in Artherosclerosis; **3)** antigen presentation; caspase cascade in apoptosis **4)** mRNA metabolism (transcription, translation, splicing), viral messenger mRNA synthesis **5)** visual perception; vitamin A and retinol metabolic process; glucose homeostasis; regulation of insulin secretion **6)** Endoderm development **7)** Glycosphingolipid Biosynthesis—Neolactoseries **8)** protein targeting to Golgi **9)** Glycolysis and gluconeogenesis; GTP metabolism **10)** unsaturated fatty acid metabolism
sAPPβ	154(102)	3608	**1)**Pancreas development; maturity onset diabetes of the young; cells; brain development; neuron fate commitment; cell fate commitment; skeletal development; **mesoderm development (EPHA1, MEF2C)**; axis specification;; hemopoietic progenitor cell differentiation; **2)** male sex differentiation; sex determination; response to osmotic stress **3) defense response (CR1, CLU, CD33, TREM2)**; TREM 1 signaling; type I interferon production; cytokine secretion; MyD88-dependent toll-like receptor signaling; immunoglobulin mediated immune response; B-cell mediated immunity; leukocyte migration; adipocytokine signaling; caspase cascade in apoptosis; DNA deamination; superoxide release **4)** regulation of transcription in response to stress; porphyrin catabolism; Calcineurin NFAT-dependent transcription in lymphocytes **5)** Blood coagulation (PTK2B); Plug formation **6)** memory **7)** gamma-aminobutyric acid transport **8) transcription (MEF2C)**; translation; splicing; **HIV infection and production (DSG2)**; **9)** dATP/dITP metabolism **10)** Golgi to ER retrograde transport; **vesicle coating (PICALM);** vesicle targeting; Transport_RAB1A regulation pathway; Caveolar-mediated Endocytosis; **membrane budding (PICALM)**; pancreatic juice secretion
Total	372(208)	6347	

**Fig 1:**
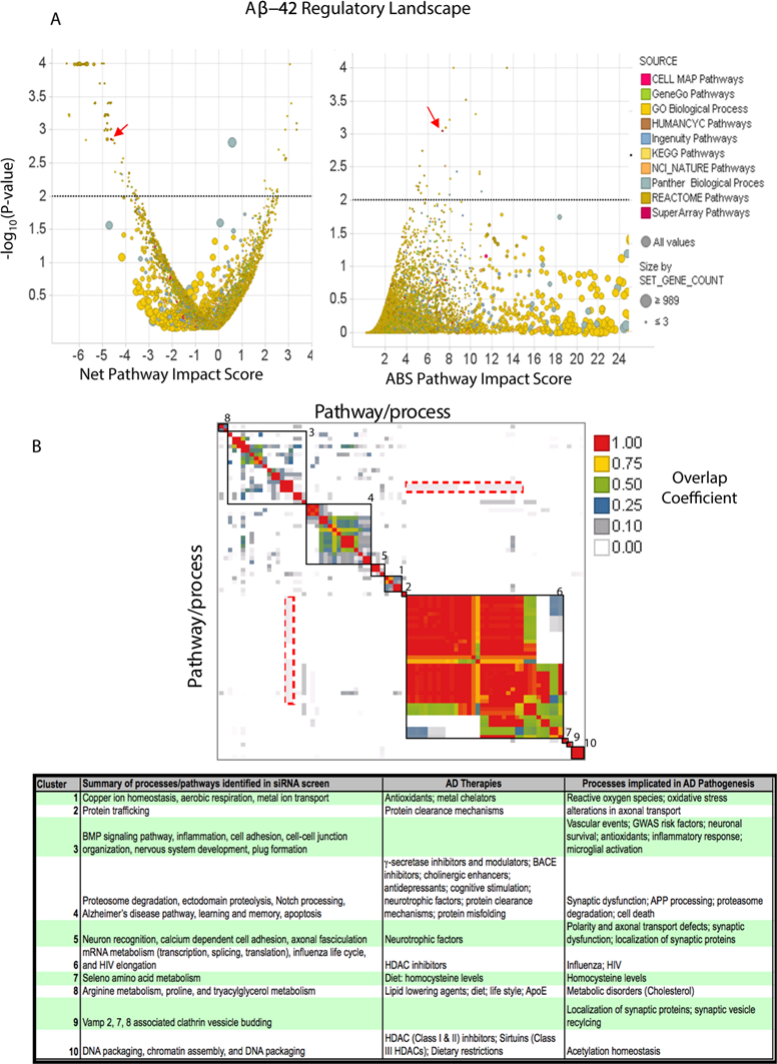
Identification of pathways that regulate APP processing (Aβ42). A. By combining the *P*-value and PI score, we identified pathways/processes that, when knocked down, significantly affect the readout in question. Depicted here are the results for Aβ42 readout. Each circle represents a process/pathway set and the size of the circle corresponds to the number of genes, measured in the screen, that comprise each pathway. Colors correspond to the database from which the pathway/process set was derived. Y-axis represents the likelihood of a pathway of a given size to have the corresponding net or absolute PI score by chance. Black dotted line corresponds to p-value = 0.01 or -log_10_(p-value) = 2. One of the most significant sets was the AD pathway as defined by KEGG (red arrow). This pathway contains γ-secretase, β secretase, and other enzymes known to either cleave APP or degrade Aβ42. B. Clustering of candidate pathways/processes based on gene overlap. The overlap between two pathways/processes is determined by the ratio of the overlap of the smaller with the larger set to the size of the smaller set (see materials and methods). Clusters (black boxes) of highly overlapping pathways/processes were identified using hierarchical clustering. Cluster 4 contains the AD pathway. This type of representation also allows for the identification of interplay across the different pathways/processes. For example, the red-dashed squares indicate overlap between sets in Cluster 3 (inflammation and cell adhesion) with genes in Cluster 6 (mRNA processing, translation, and transcription). The table captures each cluster which consists of pathways/processes that share similar overlapping patterns. Several of these pathways/processes have been implicated in modulating γ-secretase activity, have been implicated in AD pathogenesis, and/or are under consideration as strategies for the treatment and prevention of AD [1,3,7,24].

Pathway/processes identified as regulating each readout are listed (see supplementary information for full list) and organized based on cluster membership. Each cluster corresponds to pathway/processes sets that have a high degree of overlap (i.e. share common genes) (see Fig. 1B). Most of the pathway/processes listed here are consistent with factors known to play a role in the pathogenesis of Alzheimer’s disease (see supplementary information)[25]. Number of gene sets (pathways/processes) identified as significant for each readout at P < 0.01. * The number in parentheses indicates the number of unique gene sets after merging identical gene sets based on size and composition; some gene sets are identical and only differ in how they are named (see S12 Supplementary Information). In bold are pathway/process sets that contain at least one gene (in parentheses) found to be significant in AD GWAS studies [21,22,25]. In italics correspond to pathways/processes directly associated with APP processing.

### Identifying the “landscape” of APP processing: pathways that regulate Aβ42 levels

In total, 82 unique pathways/process were identified from either Net or ABS PI scores (Fig. 1; Table 1). Fig. 1 summarizes the main biological processes and pathways that regulate Aβ42 production. One of the most significant regulators of Aβ42 levels was the “Alzheimer's disease pathway” (Net PI -4.66, *P* = 0.0014; ABS PI 7.37, *P* = 0.0009) as defined by the KEGG database (Fig. 1, Table 1)[26]. This pathway contains APP itself, the γ-secretase complex, β-secretase enzymes (BACE1 and BACE2), as well as other enzymes known to either cleave APP or degrade Aβ42, such as insulin degrading enzyme (IDE) and neprilysin[27]. Note that not all the genes in the pathway have a significant Z* score (e.g. >+2 or < -2), despite the fact that many of these genes (e.g. IDE, neprilysin) are involved in either the production or degradation of Aβ42 (S6 Supplementary Information). However, since we are considering the whole pathway, as opposed to just individual genes, these genes are not excluded from further consideration.

Other significant gene sets that are consistent with the expected biology include the following: “Notch receptor processing and trafficking”; “membrane protein ectodomain proteolysis” (the general mechanism involved in processing type I transmembrane proteins such as APP); and “Presenilin action in Notch and Wnt signalling”. Some of the most significant pathways were those associated with gene transcription, mRNA splicing, and protein translation. These observations are also consistent with biological expectations that: (1) knock-down of APP and its corresponding proteolytic enzymes would reduce Aβ42 production; and (2) knock-down of a gene in transcription/translation related processes would result in lower levels of protein production, and hence Aβ42[20]. Such biological consistency can also be observed for other readouts. For example, most processes known to affect Aβ42 also affect Aβ40 (see below), which is expected given that both these peptides are produced as a result APP proteolysis by the same enzymatic complex (S7 Supplementary Information).

Since proteins can participate in more than one biological process/pathway, and because there are similarities and differences in how databases define pathways, we derived a matrix representing all vs. all overlap of these gene sets in order to identify redundancies as well as cross-talk across Aβ42-regulatory processes (Fig. 1B). The overlap matrix was then clustered to identify highly overlapping pathways and processes (see Materials & Methods). The clustering procedure produced ten clusters of overlapping pathways/processes, and revealed the regulatory landscape of Aβ42 (Fig. 1B, Table 1). Table 1 provides a description of the main themes for each cluster. The landscape also illustrates the cross-talk across distinct cellular processes where, as expected, sets such as “neuron recognition” and “axonal fasciculation” overlap with “memory”, but also overlap with “calcium-dependent cell adhesion” and “immunity and defence” pathways. Another example is the overlap between “cell junction and maintenance” with processes such as “mRNA editing” and “gene transcription”. Cluster 4 contains several processes known to regulate APP processing (Fig. 1B). Several of these pathways and processes have been implicated in AD pathogenesis and cover most strategies under consideration for the treatment and prevention of AD (Fig. 1B) as well as several candidate risk factors identified in GWAS studies (Table 1; S8 Supplementary Information)[1,3,7,9,24].

Other pathways and processes were also identified. Examples include "Integrin-mediated signalling", "neuron recognition", "BMP signalling pathway", "Arginine and proline metabolism", and "Lectin pathway of complement activation" (see S4 and S5 Supplementary Information). Links between some of these pathways/processes and AD and amyloid have been reported previously [28–36]. However the specific molecular mechanisms of how these processes are linked to AD have not been fully determined. Our results suggest that these processes regulate Aβ42 biology.

### Differential impact of pathways/processes on biological endpoints

**Fig 2:**
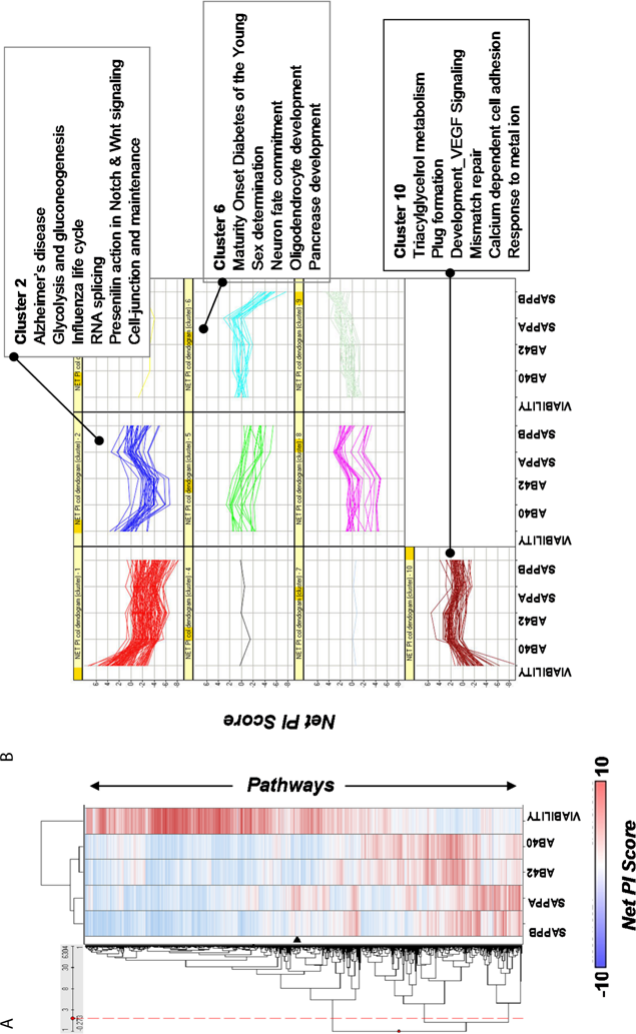
Differential effects of pathways on different readouts. Not all pathways, if knocked down by siRNA, affect biological endpoints in the same manner. A. The dendrogram on the left represents hierarchical clustering of pathways across different readouts using their Net PI score. Each row corresponds to a pathway. Blue: negative PI score (readout decreased). Red: positive PI score (readout increased). B. Individual pathway/process profiles across the readouts for each cluster. This representation allows one to identify pathways/processes that may have favourable profiles (lower net levels of amyloidogenic peptides), such as Cluster 2 and Cluster 6, and those with undesirable profiles (greater net levels of amyloidogenic peptides), such as Cluster 10. Cluster 2 and Cluster 6 show reduction in the amyloidogenic peptides Aβ40, Aβ42, and sAPPβ, with increases in sAPPα (β-secretase-inhibition profile) and no net decrease in viability. Conversely, Cluster 10 pathways have strong net decreases in viability and net increases in amyloidogenic peptides, and hence could be potentially considered pathological.

APP processing is mediated by different enzymes and therefore, it is plausible that regulation of these enzymes, and subsequently APP processing, could result from activation/de-activation of different pathways. We clustered the pathways/processes using Net PI scores in order to identify differential and similar patterns of regulation. Fig. 2 illustrates how some pathways differentially regulate viability and APP proteolytic products (see S9 Supplementary Information).

#### Differential regulation of Aβ42 vs. Aβ40 peptides

Selective lowering of Aβ42 peptides over Aβ40 can be achieved by modulating the γ-secretase complex pharmacologically or via transient protein—protein interactions without affecting cleavage of the Notch protein[4,9,37,38]. Therefore, further understanding of biological mechanisms that selectively regulate the production of Aβ42 over Aβ40 is of interest.

**Fig 3:**
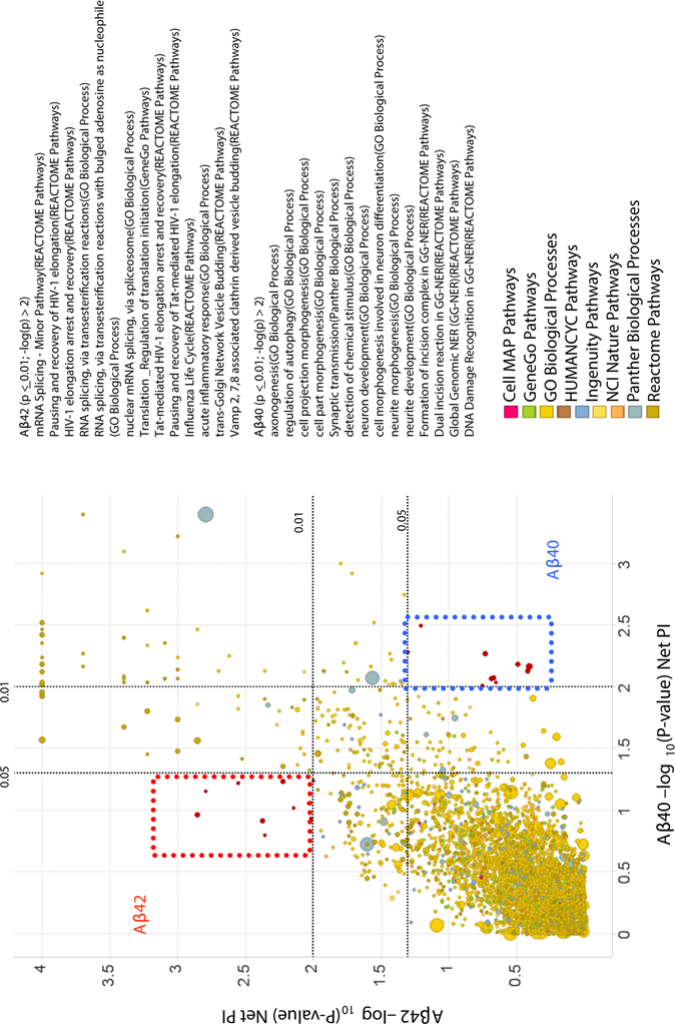
Pathways/processes that differentially regulate Aβ42 vs. Aβ40 production. A. Scatter plot of -log(-*P*-values) for Net PI scores of pathways/processes for Aβ42 against that for Aβ40. Each circle represents a pathway/process. The size of the circle corresponds to the number of genes in the set. The color corresponds to the database source from which the pathway/process was derived. As expected, most pathways and processes that regulate Aβ40 also regulate Aβ42 production. However, there are some “modulator” pathways that are significant for one readout but not the other. Red square: Aβ42-regulating pathways. Blue square: Aβ40-specific pathways.

Although most processes that were significant for Aβ42 production were also significant for Aβ40, some pathways were significant for one and not for the other (Fig. 3), such as "synaptic transmission" and "Vamp 2, 7, and 8 associated clathrin derived vesicle budding". Although synaptic activity affects levels of both Aβ40 and Aβ42, knock-downs of some of the genes in this biological process have a significantly larger effect on Aβ40 levels than on Aβ42[39]. Interestingly, knock-down of Vamp8 has been shown to reduce Aβ42, but not Aβ40 levels, suggesting potential differences in membrane targeting and/or fusion of these peptides[40].

#### Differential regulation of sAPPα vs. sAPPβ production

Pathways that differentially regulate sAPPα and sAPPβ were also identified. Contrary to the production of Aβ40 and Aβ42, which are regulated by the same enzymatic complex, the production of sAPPα and sAPPβ is regulated by distinct enzymes such as the metalloproteinases TACE/ADAM10, ADAM17, and MDC-9, and the aspartyl protease BACE1, respectively[1,41]. Furthermore, production of sAPPα is mainly mediated extracellularly, and is usually associated with processes that are thought to be beneficial to the cell such as neuronal transmission[1,39,41]. Conversely, sAPPβ is produced intracellularly as BACE1 is mainly located in the late Golgi/trans-Golgi network and endosomes[41]. Consistent with the cellular location of BACE1, pathways/processes such as “Golgi-to-ER retrograde transport (REACTOME pathways)”, “retrograde vesicle-mediated transport, Golgi to ER (GO Biological Process)”, and “caveolar-mediated endocytosis (Ingenuity Pathways)” were found to be significant regulators of sAPPα but not of sAPPβ, whose production is mainly limited to the cell surface (Table 1; see S10 Supplementary Information).

In addition to cell-compartmental differences, pathways/processes such as “maturity-onset diabetes of the young (KEGG pathways)”, “adipocytokine signalling pathway (KEGG pathways)”, and processes involved in pancreas biology and development were found to be significant for the production of sAPPα but not for sAPPβ (see S10 Supplementary Information). These observations are consistent with the emerging role of insulin resistance and deficiency in AD[42].

### Pathway-based analysis facilitates identification of mechanistic link between AD and diabetes

**Fig 4:**
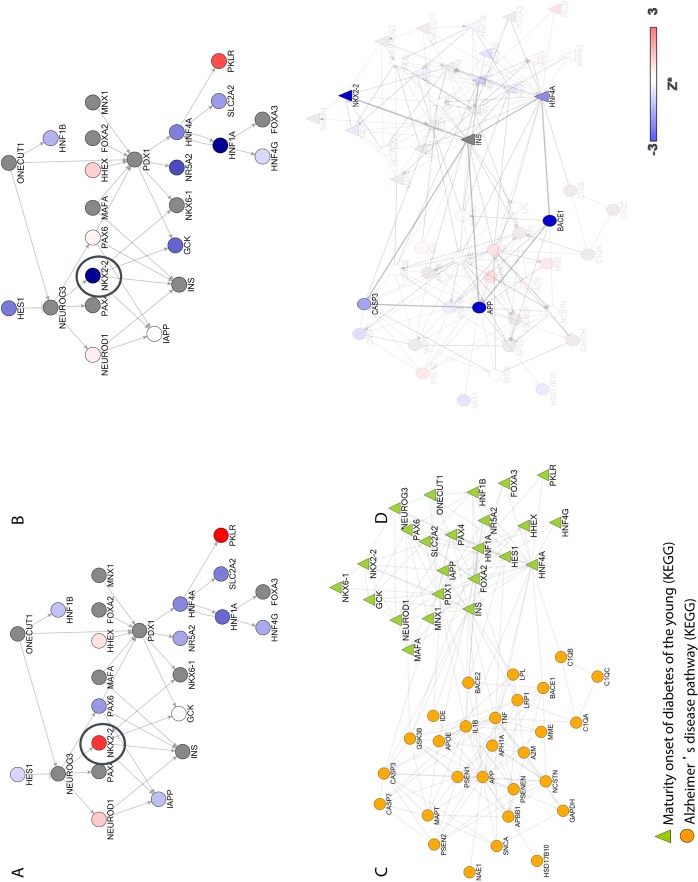
“Maturity onset diabetes of the young” pathway (KEGG) [26]. This pathway was found to be a significant regulator of sAPPβ. Proteins/genes are coloured based on their corresponding Z* values for sAPPα (A), and sAPPβ (B). Genes do not behave equally across the different readouts. For example, knock-down of NKX2–2 (black circle), which is a homeobox transcription factor, results in a significant decrease of sAPPβ (Z* = –12.3) but increases sAPPα (Z* = 2.4). Hence, the mechanism by which this pathway would favour the production of sAPPβ over sAPPα could potentially be mediated by this transcription factor. C. “Maturity onset diabetes of the young (MODY) (KEGG)” and "Alzheimer's disease" pathways (KEGG database). The network illustrates how proteins from these two pathways interact with/regulate each other. D. Two potential mechanisms by which sAPPβ levels can be lowered. One hypothetical mechanism could be via NKX2–2 regulation of APP processing via an insulin-mediated pathway. Knock-down of NKX2–2 would result in increased insulin levels leading to inhibition of caspase 3 activation and hence decreased cleavage of APP by caspase 3 at the BACE1 cleavage site[69–71]. Increased insulin levels have been associated with decreases of intracellular accumulation of Aβ levels, and caspase 3 has been shown to regulate APP processing via BACE1-related mechanisms [71–73]. Knock-down of caspase 3 in this study reduces sAPPβ levels. Although the insulin gene was not included in the screen, the knock-downs of NKX-2 and caspase 3 are consistent with known biology (i.e. reduction in levels of sAPPβ. An alternative hypothesis could be via HNF4A, a transcription factor previously characterized as binding to the BACE promoter [74]. Genes/proteins in the network are coloured by their corresponding sAPPβ Z* values.

So far, the detailed underlying mechanism by which diabetes and AD can be linked is unknown[1]. In order to understand how regulation of this pathway affects the production of sAPPβ we generated two different views of the “Maturity onset diabetes of the young” pathway, one that demonstrated how individual proteins in this pathway regulate APP processing (Figs. 4A-D) and the other that consisted of proteins from this pathway as well as the “AD pathway” (Figs. 4A-D), with the aim of determining whether proteins in the diabetes pathway would interact with/regulate proteins that have been implicated in AD.

Maturity onset of diabetes of the young, or MODY, is a monogenic form of diabetes with onset at adolescence and early adulthood. Mutations in genes in this pathway limit the ability of the pancreas to produce insulin[43]. Knock-downs of proteins involved in “Maturity onset diabetes of the young”, or MODY, have different effects on APP proteolytic products. For example, knock-down of NKX2–2, which is a homeobox transcription factor, results in a significant decrease of sAPPβ (Z* = –12.3) but increases sAPPα (Z* = 2.4). Hence, the mechanism by which this pathway would favour the production of sAPPβ over sAPPα may be mediated by this transcription factor, or potentially via other transcription factors such as HNF4A (Fig. 4D).

## Discussion

In this report we demonstrate that pathway-based analysis of genome-wide siRNA screens can be leveraged to identify key regulatory processes of different biological endpoints; in this case proteolytic processing of APP. This was achieved despite the known limitations of large-scale siRNA screens, suggesting that leveraging our knowledge of biological pathways/processes can improve our ability to interpret and leverage this technology.

Most processes/pathways identified in this study have been implicated in AD and in APP biology as well as in ageing and neuronal vulnerability [1,9,24,44] (S8 Supplementary Information). Many of the risk factors (CLU, BIN1, PICALM, CR1, CD33, EPHA1, TREM2) recently identified in several GWA studies are members of processes/pathways found to be significant for at least one of the readouts including 5 candidate genes (PTK2B, SORL1, DSG2, INPP5D, MEF2C) from newly identified loci based on meta-analysis (see Table 1)[21,22,25,45]. In other words, almost 50% of the risk factors identified in GWAS studies are members of the processes identified in our work. For example, Clusterin (CLU or APOJ) and CR1 (complement component 3b/4b) are members of the “acute inflammatory response” biological process, which has been found to impact Aβ42 production significantly (Fig. 2, Table 1). Wakabayashi *et al*. demonstrated how proteins involved in vesicle trafficking, adhesion, and integrin signalling affect the levels of Aβ40 and Aβ42 via interactions with the γ-secretase complex[40]. Such observations are consistent with our identification of “Vamp 2,7, and 8 mediated vesicle trafficking”, "integrin-signalling pathway" and “cell adhesion pathways” as regulators of Aβ42 production[40] (Fig. 2C, Table 1 clusters 3 and 9). The identification of “BMP signalling pathway” as a regulator of Aβ42 is also consistent with observations that blocking TGF-β–Smad2/3 immune signalling attenuates brain parenchymal and cerebrovascular amyloid deposits in Tg2576 mice, an animal model of AD that overproduces Aβ42[46]. Similarly, processes shown to regulate γ-secretase activity, such as synaptic transmission, inflammation, and cholesterol metabolism (triacylglycerol metabolism), were identified [1,9] (Table 1). Recently DKK1 and Wnt have been implicated in Clusterin regulation of Aβ toxicity, both of which are members of the “Presenilin action in Notch and Wnt signalling” found here to be significant regulator of Aβ42 (see Table 1)[47]. These observations provide additional supporting evidence for the role of these processes in APP biology.

In addition to identifying pathways/processes that are relevant for APP biology, we also identified pathways that differentially regulate APP processing. Although common pathways/processes were found to regulate Aβ40 and Aβ42, in some cases there was a bias of processes towards one peptide over the other, suggesting that some of the processes may modulate the γ-secretase complex and, thus, providing novel mechanisms of selective lowering of Aβ42. Differences in pathways were even more pronounced between sAPPα and sAPPβ as expected, given the different enzymes responsible for their production and differences in subcellular localization of where these peptides are produced [9]. Of interest was the identification of processes/pathways related to diabetes (such as the MODY pathway) as significant for the production of sAPPβ but not for sAPPα, consistent with the growing role of metabolic disorders in AD[1,48–52].

The MODY pathway contains several transcription factors that are critical not only for the proper development of pancreatic islet cells, but also for neuronal development[53]. There is a clear difference between the effects of knocking down NKX2.2 between sAPPα and sAPPβ suggesting a role of this transcription factor in differential regulation of APP processing. While the precise mechanism of how the MODY pathway regulates APP processing remains to be determined, by leveraging protein-protein interaction networks in conjunction with siRNA data, we can propose plausible hypotheses; this pathway may regulate APP biology either via NKX2.2, insulin, and caspase mediated mechanism or via HNF4A regulation of BACE1 (see Fig. 4 for more details). This underscores the power of pathway/process-based analysis of siRNA screens, where a biological context can be leveraged not only for the interpretation of a screen, but also in subsequent validation experiments. For example, animal models of NKX2.2 are available and so are activators and inhibitors of GCK, a glucokinase that is a member of the MODY pathway (Fig. 4)[54–56]. In other words, pathway scoring provides a top down approach of identifying the pathway/process of interest, followed by detailed drill down on potential mechanisms, as opposed to trying to build the rationale based one gene at a time.

**Fig 5:**
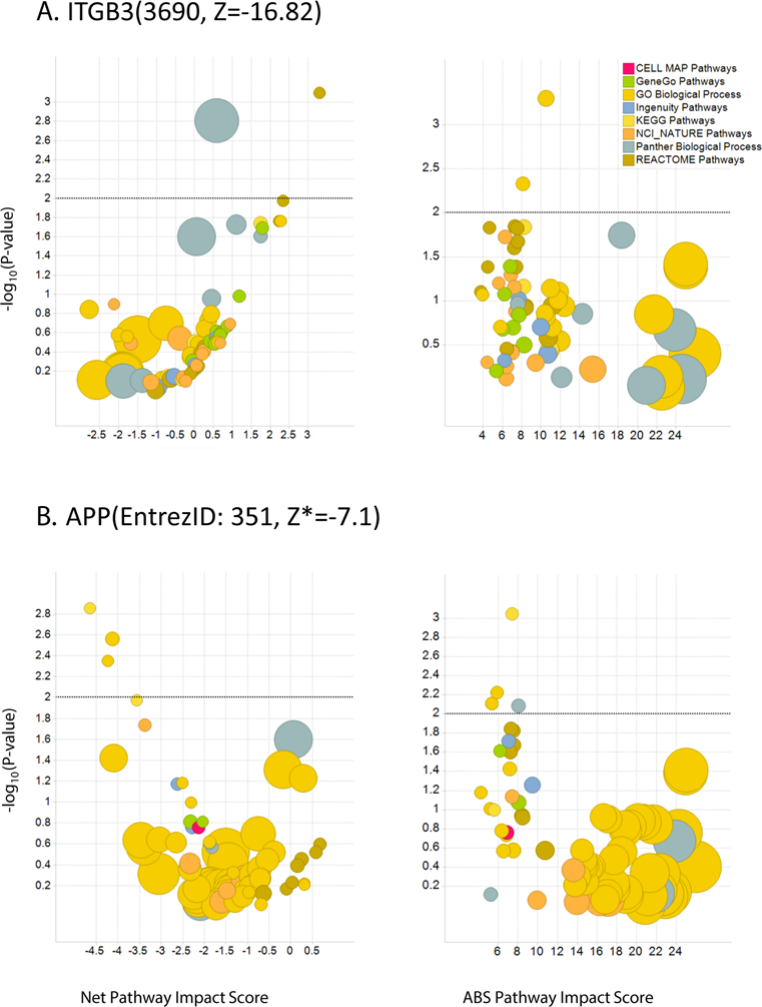
Pathway/process context matters. Not all pathways/processes that contain genes with extreme values are significant suggesting that the approach may be resistant to to outliers. For example, ITGB3 and APP are clear outliers with Z* scores of 7.18 and -7.1, respectively but not all of their corresponding pathways/processes were found to be significant regulators of Aβ42. Each circle corresponds to pathway/process and the size corresponds to the number of genes in that pathway/process. Y-axis represents the likelihood of a pathway of a given size to have the corresponding net or absolute PI score by chance. Black dotted line corresponds to p-value = 0.01 or -log_10_(p-value) = 2 and the x-axis corresponds to the either Net or ABS PI score based on the Aβ42 readout.

Selecting genes of interest based on extreme values poses a few challenges. First, it is well documented that off-target effects plague the interpretation of siRNA screens, where the effect of an siRNA probe on the biological endpoint is a result of down-regulation of the off-targets as opposed to the initially intended target [57]. Many notable efforts have been made in developing methods to address these issues [58–61]. Secondly, even in the event that off-target effects were not an issue, siRNA probes are not equally potent; genes may have a small effect on the endpoint due to compensatory mechanisms, and protein stability is not uniform across siRNA targets. For these reasons, we focused on prioritizing pathways/processes as opposed to individual genes. We assume that the off-target effects for each siRNA probe/gene are independent and that there is a very small likelihood of siRNA probes, targeting different genes in the same pathway, to have the same off-target effects that would contribute to the regulation of the same phenotype (e.g., lowering levels of Aβ42). Hence, the permutation analysis can test whether the relationship of genes in a pathway/process in aggregate should have a larger effect than when no relationship is present; that is, they do not work in concert in the same pathway or process. In fact, not all gene sets that contained genes with extreme values were significant, suggesting that context is important in determining significance (Fig. 5). Furthermore, we were able to rescue false negatives by focusing on the pathway level (see S6 Supplementary Information).

The agreement between our results, human genetic studies, and the literature supports the idea that a pathway-based analysis of whole-genome siRNA screens can be used to map the regulatory landscape of different biological endpoints. Pathways/processes in such a landscape can be categorized as follows: (1) direct mechanisms (e.g. γ-secretase complex); (2) related but not direct regulatory mechanisms (e.g. other signalling pathways that regulate APP cleaving enzymes, feedback mechanisms, vesicle trafficking of soluble APP proteolytic products, and protein degradation); and (3) general cellular mechanisms that are likely to impact any readout (e.g. protein translation, gene transcription, and mRNA splicing. Although these mechanisms may seem distinct, proteins can participate in different processes in the cell. To this end we derived a relationship view of their cross-talk facilitating the interpretation of how these processes are inter-related (see S11 Supplementary Information).

We hope that the pathways/processes identified herein, and our derivation of their relationship to one another, provides a comprehensive systems biology view of APP biology that will serve as a useful resource for the AD community to further dissect the role of APP physiology, the implications of other pathophysiologies in AD, and to help identify novel Aβ lowering strategies.

## Materials and Methods

### APP siRNA screen and data processing

The siRNA screen used in this study is described in Majercak *et al*.[20]. Briefly, the screen uses HEK-293 cells, a kidney derived cell line, stably expressing a mutant form of APP that contains a four amino-acid modification (NFEV) designed to enhance cleavage by the BACE1 enzyme (S7 Supplementary Information)[20]. These cells were transfected with different siRNA pools (*n* = 3 per gene). Conditioned media from these cells were then removed after 48 hrs following incubation with siRNA pools. Aliquots were used to detect different APP proteolytic products: Aβ40, Aβ42, sAPPα, and sAPPβ. In addition, cell viability was determined by incubating cells for 2 hrs with Alamar blue.

Raw intensities from the original screen were re-processed in order to achieve the following: (1) to normalize all peptide-related values (Aβ40, Aβ42, sAPPα, and sAPPβ) to viability in order to account for differences in peptide levels that may result from differences in cell number; and (2) to account for plate-edge effects that were observed in the original screen. The original screen[20] reported values as percent activity, which is defined as the percent effect of knocking down a gene relative to a reference control. We introduced a more effective measure (denoted Z*) to represent the values for each siRNA pool. Let *Y*_*i*_ be the measured activity for a given well (log intensity), ${\tilde{Y}}_{N}$the median value of all samples, *N*, in the plate, and *MAD*_*N*_ the median absolute deviation[12] of measured values. The Z* score is defined as

$$Z*={\frac{Y_{i}-{\tilde{Y}}_{N}}{\sqrt{2}\times MAD_{N}}}_{.}$$

The original screen used 15,200 siRNA pools, some of which contained siRNA probes for which a corresponding gene could not be assigned, and/or had multiple replicates for the same siRNA pool; this pool was being used as an internal control. We removed all probes that could not be assigned to a gene. Furthermore, for every gene with multiple siRNA pools, the averaged Z* value was used. In total, 14,603 siRNA pools, representing 13,537 unique genes, were considered for analysis.

### Pathway/process data-set collection and representation

We compiled several gene sets representing pathways and biological processes from the following commercial and public databases (S2 Supplementary Information): NCI Nature Pathways[62], KEGG[26], Ingenuity (Ingenuity Systems, www.ingenuity.com), Reactome [63], GeneGo Metabase (www.genego.com), Panther Biological Processes[64], and Gene Ontology Biological Process[65]. These sources represent a list of human-curated pathways and biological processes. Pathway sets from Reactome and NCI Nature Pathways were retrieved using the cPATH [66] tool from the Pathway Commons website; only pathways that contained at least one gene used in the siRNA screen were considered. For each given pathway/process set, only those genes used in the siRNA screen were retained for the analysis. As a result the size of the processed gene set corresponds to the intersection of the genes in the original set and those monitored in the screen. Sets containing <3 genes and >1,000 genes were also excluded. The upper bound is an arbitrary cut-off indicating that sets of >1,000 genes are unlikely to yield meaningful biological interpretation. In total, 6,154 gene sets representing pathways and/or biological processes were used, with 95% (12,859) of all genes in the screens being assigned to at least one set. All the gene sets used in the screen can be found in the S2 Supplementary Information as well as in the TargetMine system (http://targetmine.nibio.go.jp/applandscape/) [67].

### Pathway impact (PI) scores and simulation *P*-value calculations

PI scores are calculated as follows:

$$\text{Net PI}=\;\frac{\overset{n}{\underset{i=1}{\sum }}Z_{i}^{*}}{\sqrt{n}}\text{ or Absolute }\left(\text{ABS}\right)\text{PI}=\;\frac{\overset{n}{\underset{i=1}{\sum }}abs\left(Z_{i}^{*}\right)}{\sqrt{n}}$$

where *n* is the number of genes in a pathway/process set, and Z*_i_ is the Z* score for each individual gene in that pathway/process set. The ABS PI score is calculated to mitigate against the scenario in which small PI scores could result from equal but opposing effects of genes in a given pathway/process.

The probability *P* of obtaining by chance a PI score equal to or greater than the observed one for a given set of size *n* was calculated as follows: the Net or Absolute PI score was computed for 10,000 random selection of *n* genes from the screen in order to derive the null distribution for each set size. Then, the probability *P* was determined by the proportion of random PI scores that were equal to or more extreme than the observed PI score. Pathway/process sets were considered significant if *P ≤* 0.01 for either the Net or ABS PI scores for each readout, respectively. Volcano plots for the results were created using Spotfire Decision Site 9.1 (http://spotifire.tibco.com); PI scores for Net or ABS values are reflected in the *x*-axis and *P*-values are represented on the *y*-axis as -log_10_(*P*-value) and hence the cut-off is -log_10_ 0.01 = 2.

### Derivation of the overlap matrix

In order to identify the extent of overlap between different gene sets, we calculated the overlap coefficient *O*_*c*_ between two sets *i* and *j* as follows:

$$O_{c}\left(i,j\right)=\left|S_{i}\cap S_{i}\right|/\left[\text{min}\left(\left|S_{i}\right|,\left|S_{j}\right|\right)\right]$$

where $\left|S_{i}\cap S_{i}\right|$ is the number of genes shared by the two sets *S*_*i*_ and *S*_*j*_, and $\text{min}\left(\left|S_{i}\right|,\left|S_{j}\right|\right)$ is the smaller size of the two sets considered[68]. *O*_*c*_ was then used to create a symmetric overlap matrix of all-versus-all set comparisons. An overlap of 1 means either: (1) the sets are identical in size and composition; or (2) the smaller set is a true subset of the larger set; conversely, an overlap of 0 means that the sets share no genes. The overlap was calculated based on the composition of gene sets (pathways/processes) with respect to the screen. Hence, the degree of overlap between two gene sets in this study may differ from the overlap of these sets when considering the complete membership for each set.

### Identification of highly overlapping pathway/process clusters

The overlap coefficient matrix *O*_*c*_ was re-ordered to identify clusters that represented related pathways/processes. Briefly, rows and columns of the matrix were re-ordered by first calculating a distance matrix based on cosine-correlation followed by average linkage clustering [68]. A matrix view was generated using the values from the *O*_*c*_ matrix and ordering from the clustering run. The generation of the cosine-correlation distance matrix and average linkage clustering were performed using the pdist and linkage functions, respectively, in Statistic Tool Box of Matlab 7.4.0 (www.mathworks.com). The matrix was plotted using Spotfire Decision Site 9.1 (www.tibco.com).

## Supporting Information

S1 Supplementary Information Describes in more detail the rationale behind both Net and PI scores. (DOCX)

S2 Supplementary Information File contains all the gene sets used in this study. The gene set name, number of genes, source (e.g. Reactome), and corresponding members (symbols, Entrez gene ids) is provided. Note that the gene set composition used in this study may not match current pathway definitions. One reason is that pathway definitions change with time and some pathways may now contain additional members. Secondly, only genes for which siRNA data is available were considered; in a scenario where the pathway contains 10 genes, but only 5 were screened, the pathway is consider to have a set size of 5.
(XLS)

S3 Supplementary Information File contains the results of the siRNA screen (i.e. Z* values) for each gene and readout. (XLS)

S4 Supplementary Information PI scores and corresponding p-values for each pathway/process set across all readouts. (XLS)

S5 Supplementary Information Significant pathways/processes subdivided per readout (viability, Aβ40, Aβ42, sAPPα, and sAPPβ) separated into different worksheets in excel. (XLS)

S6 Supplementary Information Alzheimer’s disease pathway as defined by the KEGG database at the time the analysis was performed. The pathway contains only those genes that were present in the screen.
(DOCX)

S7 Supplementary Information Figure representing the endoproteolysis of APP by different enzymes. (DOCX)

S8 Supplementary Information Summary of key biological processes associated with Alzheimer’s disease. (DOCX)

S9 Supplementary Information Cluster membership for each pathway/processes for Fig. 2 as well as in the pathway overlap matrix for each readout (see also S10 Supplementary Information). (XLS)

S10 Supplementary Information Pathways/processes found to differentially regulate sAPPα and sAPPβ. (DOCX)

S11 Supplementary Information Regulatory landscapes for all the readouts. (DOCX)

S12 Supplementary Information File contains gene sets that were merged based on having identical composition. That is, genes that contain exactly the same genes are merged into one.
(TXT)
