# Correction: Pathway-Based Analysis of Genome-Wide siRNA Screens Reveals the Regulatory Landscape of App Processing

**DOI:** 10.1371/journal.pone.0129641

**Published:** 2015-06-01

**Authors:** Luiz Miguel Camargo, Xiaohua Douglas Zhang, Patrick Loerch, Ramon Miguel Caceres, Shane D. Marine, Paolo Uva, Marc Ferrer, Emanuele de Rinaldis, David J. Stone, John Majercak, William J. Ray, Chen Yi-An, Mark S. Shearman, Kenji Mizuguchi

Fig 2, Fig 3, and Fig 4 incorrectly appear sideways. Please see the corrected Fig 2, Fig 3, Fig 4 here.

**Fig 2:**
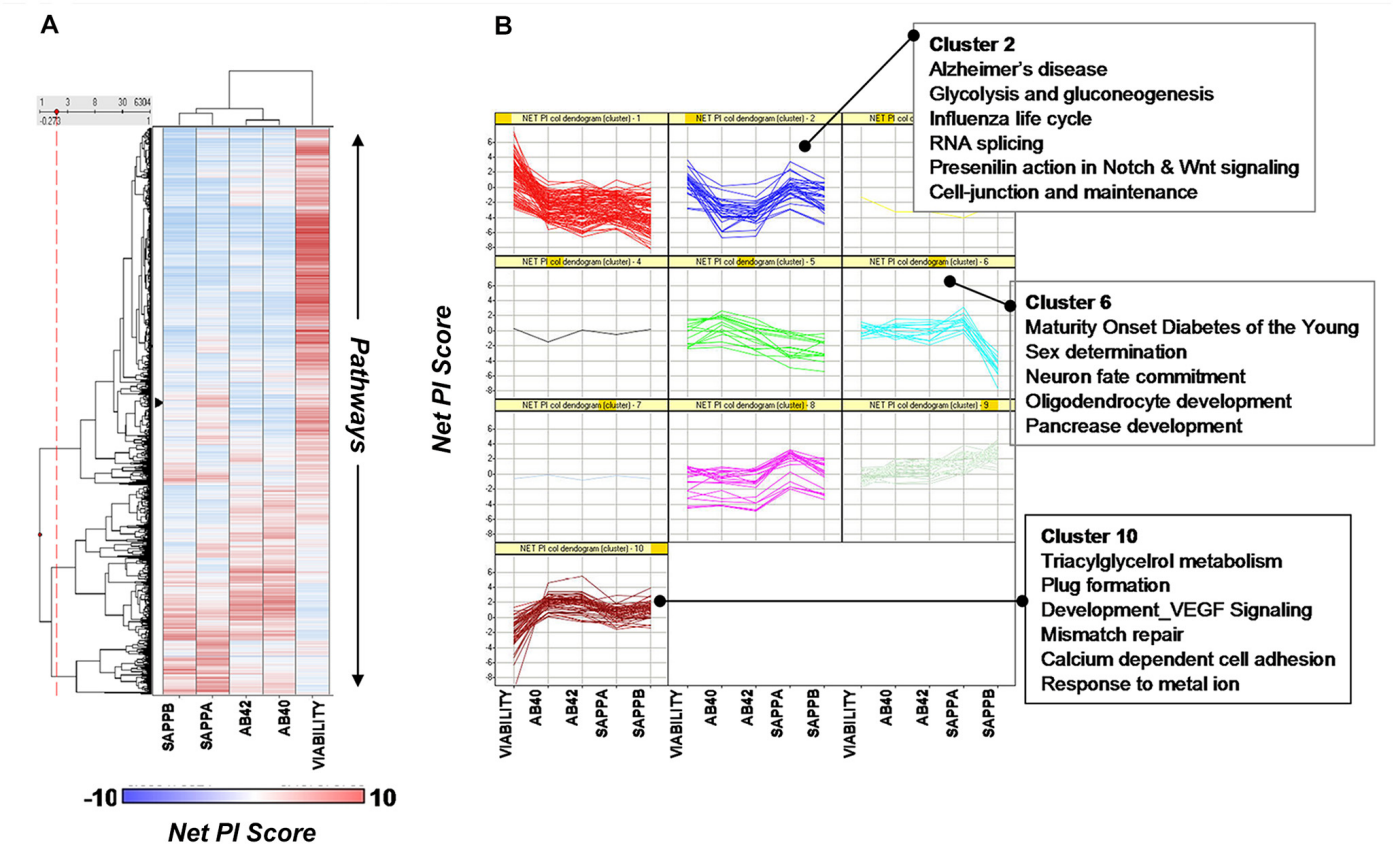
Differential effects of pathways on different readouts. Not all pathways, if knocked down by siRNA, affect biological endpoints in the same manner. A. The dendrogram on the left represents hierarchical clustering of pathways across different readouts using their Net PI score. Each row corresponds to a pathway. Blue: negative PI score (readout decreased). Red: positive PI score (readout increased). B. Individual pathway/process profiles across the readouts for each cluster. This representation allows one to identify pathways/processes that may have favourable profiles (lower net levels of amyloidogenic peptides), such as Cluster 2 and Cluster 6, and those with undesirable profiles (greater net levels of amyloidogenic peptides), such as Cluster 10. Cluster 2 and Cluster 6 show reduction in the amyloidogenic peptides Aβ40, Aβ42, and sAPPβ, with increases in sAPPα (β-secretase-inhibition profile) and no net decrease in viability. Conversely, Cluster 10 pathways have strong net decreases in viability and net increases in amyloidogenic peptides, and hence could be potentially considered pathological.

**Fig 3:**
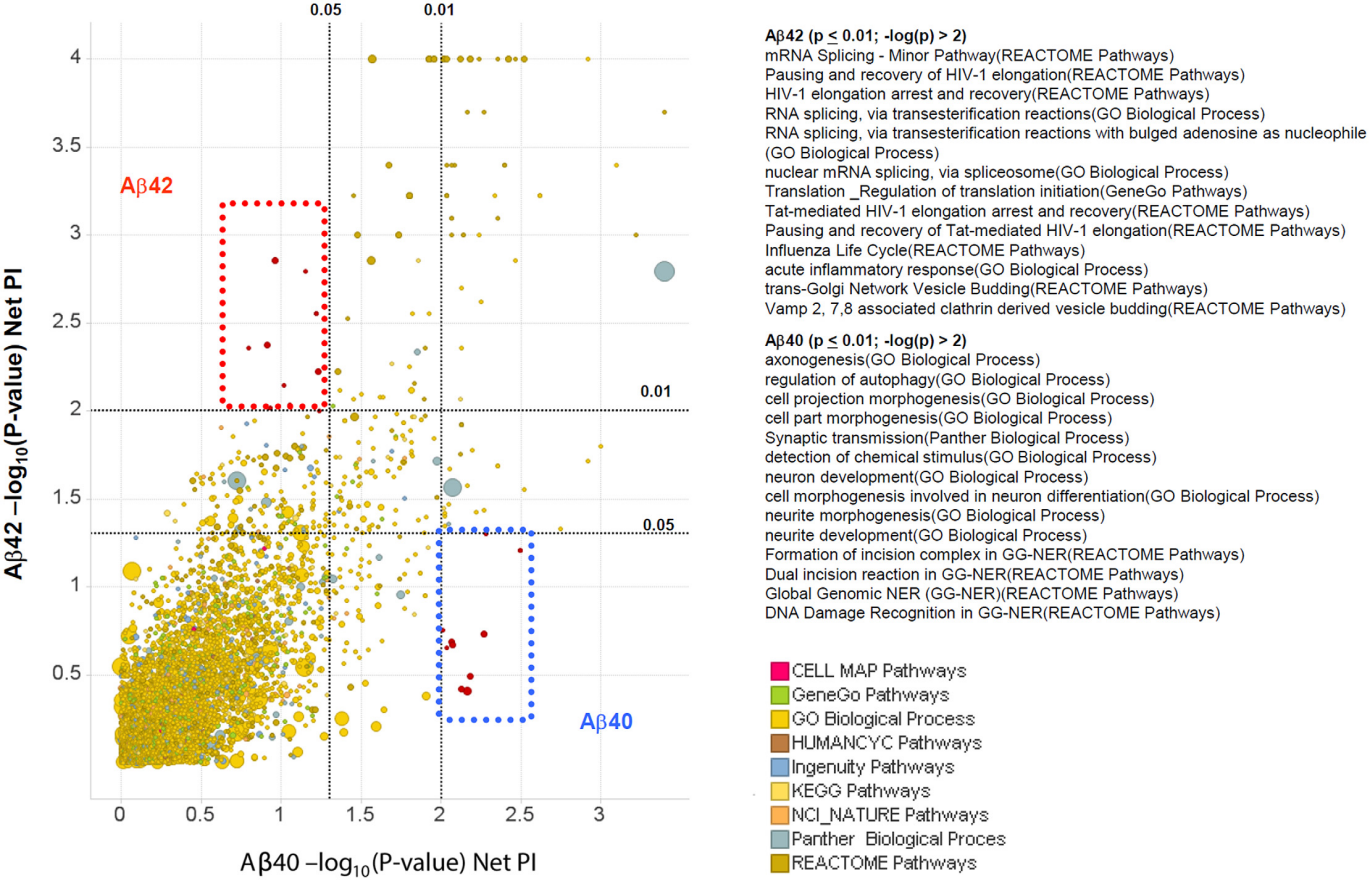
Pathways/processes that differentially regulate Aβ42 vs. Aβ40 production. A. Scatter plot of-log(-P-values) for Net PI scores of pathways/processes for Aβ42 against that for Aβ40. Each circle represents a pathway/process. The size of the circle corresponds to the number of genes in the set. The color corresponds to the database source from which the pathway/process was derived. As expected, most pathways and processes that regulate Aβ40 also regulate Aβ42 production. However, there are some “modulator” pathways that are significant for one readout but not the other. Red square: Aβ42-regulating pathways. Blue square: Aβ40-specific pathways.

**Fig 4:**
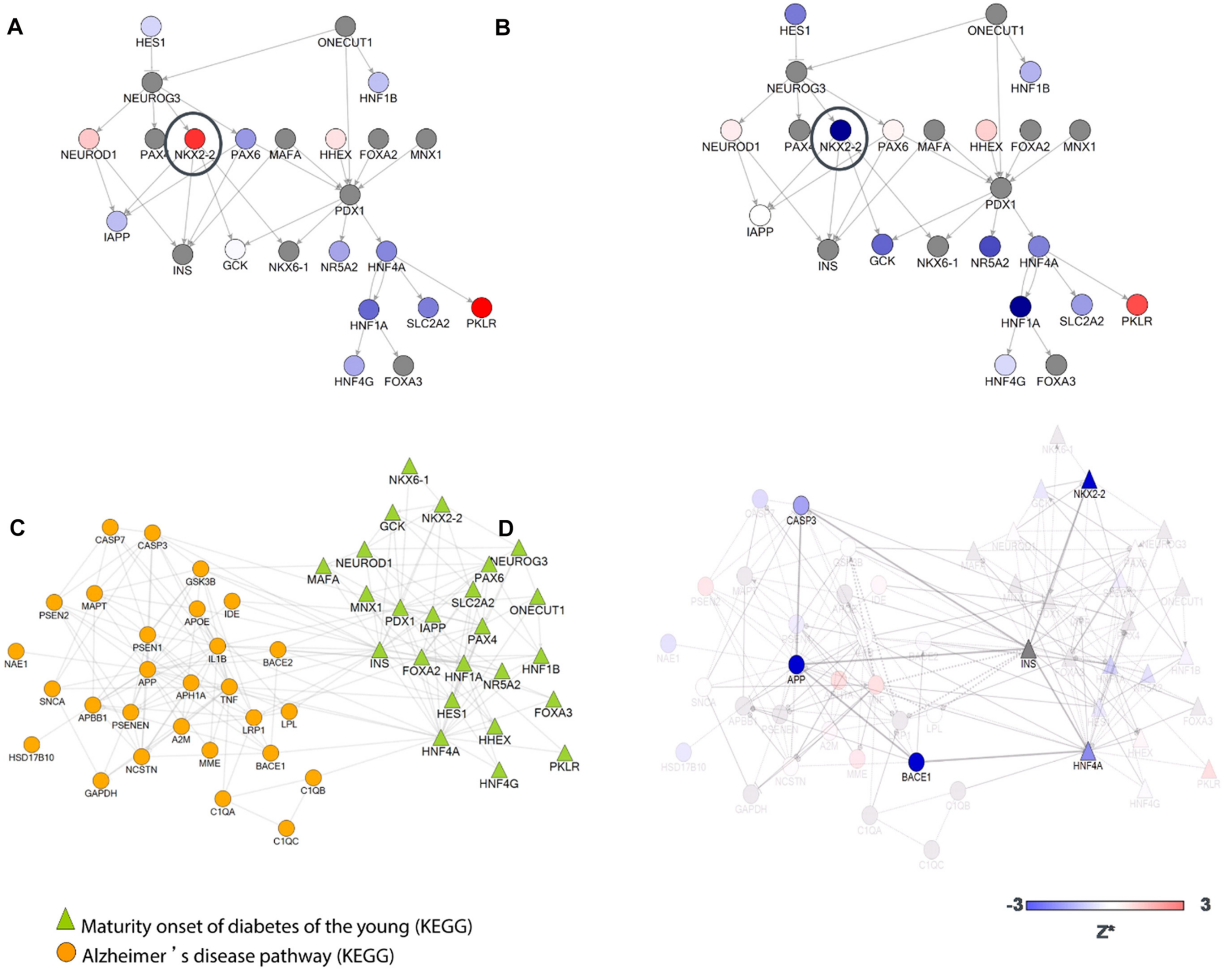
“Maturity onset diabetes of the young” pathway (KEGG) [26]. This pathway was found to be a significant regulator of sAPPβ. Proteins/genes are coloured based on their corresponding Z* values for sAPPα (A), and sAPPβ (B). Genes do not behave equally across the different readouts. For example, knock-down of NKX2-2 (black circle), which is a homeobox transcription factor, results in a significant decrease of sAPPβ (Z* = –12.3) but increases sAPPα (Z* = 2.4). Hence, the mechanism by which this pathway would favour the production of sAPPβ over sAPPα could potentially be mediated by this transcription factor. C. “Maturity onset diabetes of the young (MODY) (KEGG)” and "Alzheimer's disease" pathways (KEGG database). The network illustrates how proteins from these two pathways interact with/regulate each other. D. Two potential mechanisms by which sAPPβ levels can be lowered. One hypothetical mechanism could be via NKX2-2 regulation of APP processing via an insulin-mediated pathway. Knock-down of NKX2-2 would result in increased insulin levels leading to inhibition of caspase 3 activation and hence decreased cleavage of APP by caspase 3 at the BACE1 cleavage site[69–71]. Increased insulin levels have been associated with decreases of intracellular accumulation of Aβ levels, and caspase 3 has been shown to regulate APP processing via BACE1-related mechanisms [71–73]. Knock-down of caspase 3 in this study reduces sAPPβ levels. Although the insulin gene was not included in the screen, the knock-downs of NKX-2 and caspase 3 are consistent with known biology (i.e. reduction in levels of sAPPβ). An alternative hypothesis could be via HNF4A, a transcription factor previously characterized as binding to the BACE promoter [74]. Genes/proteins in the network are coloured by their corresponding sAPPβ Z* values.
