# Supplementary material for: Pathway-Based Analysis of Genome-Wide siRNA Screens Reveals the Regulatory Landscape of App Processing
Source: PLoS One. 2015 Feb 27;10(2):e0115369. doi: 10.1371/journal.pone.0115369 (PMC4344212; doi:10.1371/journal.pone.0115369)
Supplement: S1 Supplementary Information — (DOCX) [file pone.0115369.s001.docx]

**Rationale for Net and Absolute PI scores**

Pathway/processes may contain both “activators” and “inhibitors” of the biological endpoint. Therefore, it is of interest to determine whether or not perturbing such a pathway will tend towards an inhibitory or an activation effect. For this reason a net score is calculated. We use the absolute score to determine whether or not the pathway/process affects the endpoint irrespective of the direction of the magnitude. The absolute score also accounts for pathways/processes that have a low net value due to the presence of activators and inhibitors that exhibit effects on the endpoint of similar magnitude but that cancel each other out.

A

B

Absolute PI rescues pathways with low net values due similar distribution and magnitude of “acitvators” and “inhibitors” within the same pathway. **A**. Scatter plot of Net vs. ABS PI scores. Each circle corresponds to a pathway/process. The size of the circle corresponds to the number of genes in the set. Dashed box highlights gene sets with low Net PI scores. **B.** Scatter plot of the –log10(p-value) for ABS PI (x-axis) and NET PI (y-axis). Not that the Arginine and Proline Metabolism pathway is only significant in the ABS score.

Conversely, in order to determine whether the magnitude and direction of the effect for a given pathway is of interest, a net score is calculated and a two-tailed is performed as opposed to a one-tailed test for the absolute values. The mRNA editing (REACTOME Pathways) has a strong net effect on the endpoint. There is a bias towards reduction of Aβ42 in this pathway.


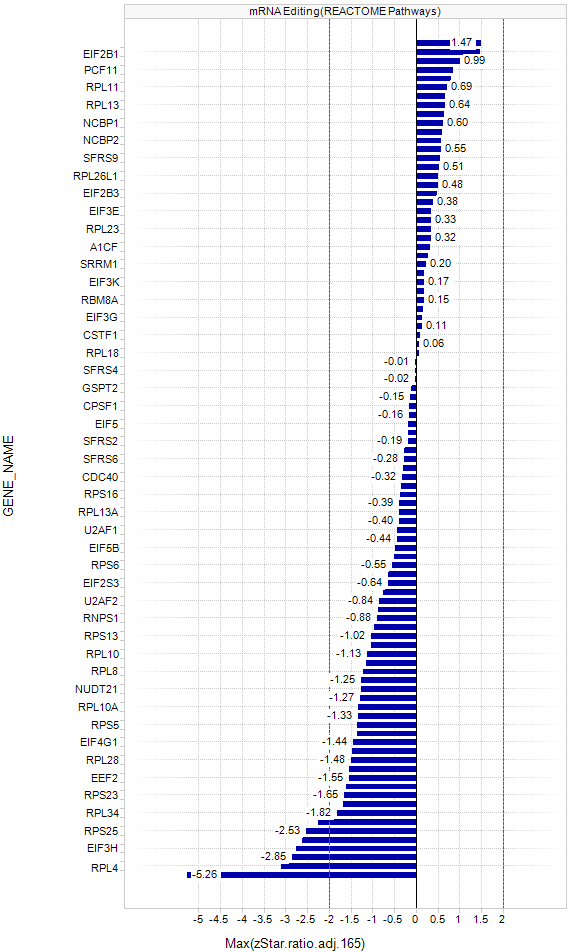


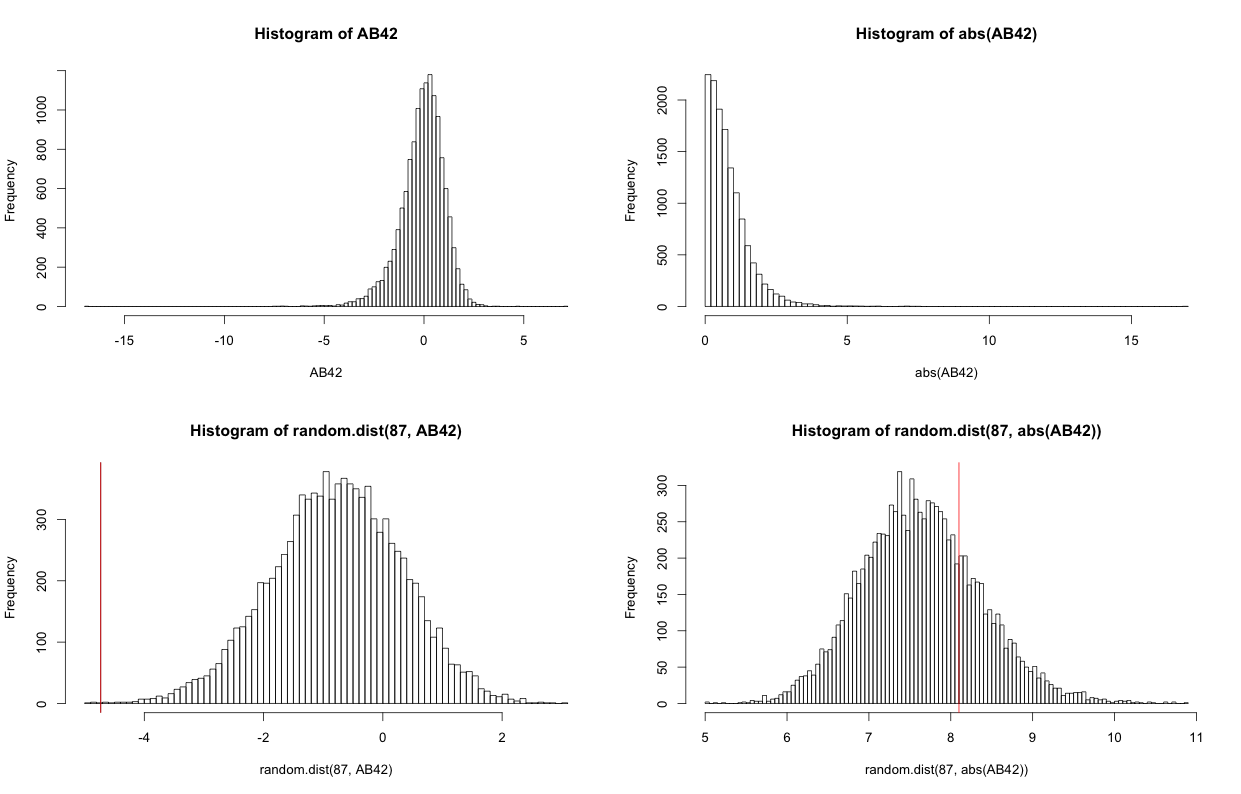


Top two plots show the distribution of Z* values for each gene with respects to Aβ42. The right top plot reflects the absolute distribution of these values. The two plots below are the random scores for a pathway/process that contains 87 genes. The Net PI score for mRNA editing pathway is -4.73 and this is an extreme value unlikely to occur by chance (red line in bottom left plot). Conversely, the absolute PI score (ABS PI) for this pathway is 8.10 (red line) and in the distribution of random gene set scores it is not an extreme value (bottom right plot).
