# Supplementary material for: Pathway-Based Analysis of Genome-Wide siRNA Screens Reveals the Regulatory Landscape of App Processing
Source: PLoS One. 2015 Feb 27;10(2):e0115369. doi: 10.1371/journal.pone.0115369 (PMC4344212; doi:10.1371/journal.pone.0115369)
Supplement: S6 Supplementary Information — The pathway contains only those genes that were present in the screen. (DOCX) [file pone.0115369.s006.docx]

**Supplementary figure AD Pathway**. AD pathway as defined by the KEGG database (contains only the genes assayed in the siRNA screen). The plot shows individual genes from the pathway and their corresponding Z* scores for the Aβ42 readout. Note that many of the genes in this pathway have a Z* value > |Z*| = 2 and, hence, not all genes in this pathway would have been considered as hits in the screen if the standard method was used (Figure 3.2). Most of these genes have been implicated in regulation levels, such as IDE, GSK3β, and CASP3 (Zhou et al., 2007). Notably, members of the γ-secretase complex PSEN2 and NCSTN (Nicastrin) would not have made the cut-off. The lack of uniform protein stability, probe efficacy, and impact due to position in a pathway are some of the reasons why these proteins would not have an extreme effect on the readout. For example, IDE is known to degrade Aβ42 (Nalivaeva et al., 2008). The data in this screen do not support this observation. However, measurements were taken 48 hrs following incubation with siRNA screens, and this time point may not align with the turnover of IDE. Levels of IDE have been shown to be stable after 24 hrs in a mouse model of AD (Nalivaeva et al., 2008). Levels of IDE are considered a risk factor in AD (Zou et al., 2010; Carrasquillo et al., 2010).

Zhou, S., Zhou, H., Walian, P. J., and Jap, B. K. Regulation of gamma-secretase activity in Alzheimer's disease. Biochemistry 46(10), 2553-2563. 3-13-2007

Nalivaeva, N. N., Fisk, L. R., Belyaev, N. D., and Turner, A. J. Amyloid-degrading enzymes as therapeutic targets in Alzheimer's disease. Curr.Alzheimer Res. 5(2), 212-224. 2008.

Carrasquillo, M. M., Belbin, O., Zou, F., Allen, M., Ertekin-Taner, N., Ansari, M., Wilcox, S. L., Kashino, M. R., Ma, L., Younkin, L. H., Younkin, S. G., Younkin, C. S., Dincman, T. A., Howard, M. E., Howell, C. C., Stanton, C. M., Watson, C. M., Crump, M., Vitart, V., Hayward, C., Hastie, N. D., Rudan, I., Campbell, H., Polasek, O., Brown, K., Passmore, P., Craig, D., McGuinness, B., Todd, S., Kehoe, P. G., Mann, D. M., Smith, A. D., Beaumont, H., Warden, D., Holmes, C., Heun, R., Kolsch, H., Kalsheker, N., Pankratz, V. S., Dickson, D. W., Graff-Radford, N. R., Petersen, R. C., Wright, A. F., Younkin, S. G., and Morgan, K. Concordant association of insulin degrading enzyme gene (IDE) variants with IDE mRNA, Abeta, and Alzheimer's disease. PLoS One 5(1), e8764. 2010.
