# Supplementary material for: Pathway-Based Analysis of Genome-Wide siRNA Screens Reveals the Regulatory Landscape of App Processing
Source: PLoS One. 2015 Feb 27;10(2):e0115369. doi: 10.1371/journal.pone.0115369 (PMC4344212; doi:10.1371/journal.pone.0115369)
Supplement: S7 Supplementary Information — (DOCX) [file pone.0115369.s007.docx]

**Endoproteolysis of the Amyloid Precursor Protein (APP)**


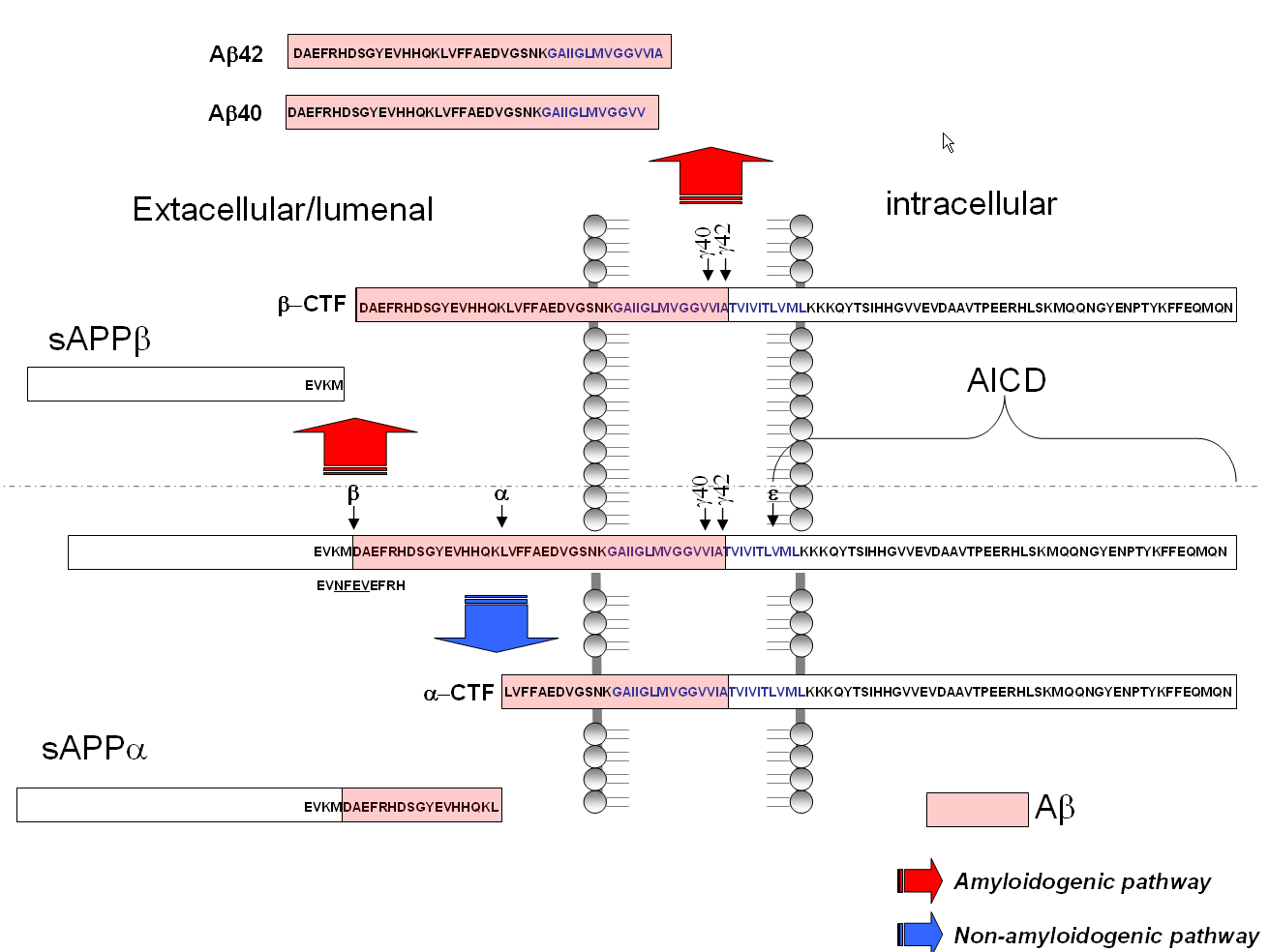


**Endoproteolysis of the Amyloid Precursor Protein (APP**). Cleavage of APP occurs at different sites and in a sequential fashion. Amyloidogenic pathway: γ-secretase cleavage of APP at the β-site producing soluble APPβ (sAPPβ) as well as a membrane bound β-CTF fragment. β-CTF is in turn cleaved by γ-secrtease first at the ε site, releasing the APP intracellular domain (AICD), followed by the γ cleavage that results in the production of Aβ40 and Aβ42. It is the inhibition of the ε -cleavage of Notch, another γ-secretase substrate, that is thought to cause gastrointestinal toxicities in the clinic. Non-amyloidogenic pathway: α cleavage of APP results in the production of soluble APPα (sAPPα). α-secretase cleaves within the Aβ domain thus precluding the generation of Aβ peptides.

Zhou, S., Zhou, H., Walian, P. J., and Jap, B. K. Regulation of gamma-secretase activity in Alzheimer's disease. Biochemistry 46(10), 2553-2563. 3-13-2007

Kopan, R. and Ilagan, M. X. Gamma-secretase: proteasome of the membrane? Nat.Rev.Mol.Cell Biol. 5(6), 499-504. 2004.
