# Supplementary material for: Pathway-Based Analysis of Genome-Wide siRNA Screens Reveals the Regulatory Landscape of App Processing
Source: PLoS One. 2015 Feb 27;10(2):e0115369. doi: 10.1371/journal.pone.0115369 (PMC4344212; doi:10.1371/journal.pone.0115369)
Supplement: S8 Supplementary Information — (DOCX) [file pone.0115369.s008.docx]

| ***Mechanism*** | **Summary of Key Evidence** |
| --- | --- |
| *Ageing* | Normal cognitive decline; synaptic loss, life style; stress life events; head trauma all contribute to risk of disease |
| *Synaptic dysfunction* | Aβ42 binds and inhibits proper synaptic function; depletion of growth factors and dysregulation of neurotransmitter systems such as acetylcholine and glutamine are observed; synaptic loss is observed with ageing |
| *Depletion of growth factors and neurostransmitter systems* | Reduced levels of neurotrophin receptors are observed in cholinergic neurons of late stage AD patients; growth factors BDNF and NGF are thought to be viable therapeutic strategies for AD showing cognitive improvements in animal models and in patients |
| *Mitochondrial dysfunction* | Aβ inhibits mitochondrial enzymes in the brain resulting in deficits in ATP production, electron transport, oxygen consumption, and mitochondrial membrane potential |
| *Oxidative stress* | Markers of oxidative stress precede pathological changes; Aβ is a potent generator of reactive oxygen and nitrogen species |
| *Insulin signalling and diabetes* | Glucose intolerance and type 2 diabetes are risk factors for dementia |
| *Vascular events* | Large incidence of ischemic disease in patients with AD; disruption of blood-brain barrier is observed in AD patients; abnormal brain Aβ clearance; head trauma is risk factor for disease |
| *Inflammation* | Activated microglia are observed in AD brains; Aβ and glial activation induce classic complement pathway; anti-inflammatory agents reported to lower risk of AD; recent GWAS studies implicate the immune system in AD (e.g. CR1, CLU); elevated levels of cytokines IL-1, IL-6, TNFα, and S100b are observed in the brain of AD patients |
| *Calcium homeostasis* | Elevated levels of intracellular calcium enhance Aβ aggregation; Presenilin mutations are associated with abnormal levels of calcium in the endoplasmic reticulum; excitatory neurotransmission via glutamate enhances calcium levels via NMDA receptors |
| *Aberrant cell cycle re-entry* | Oxidative stress, and APP proteolytic products (Aβ and C99) induce DNA replication and death in neurons; de-regulation of kinases that maintain cell-cycle exit are observed in AD. |
| *Axon transport defects* | Reduction in transport of proteins to the synapse and disruption of microtubule network due to tau hyperphosphorylation are observed in AD; transport deficits result in accumulations in Aβ, vesicle proteins, and kinesins causing axonal swelling |
| *Autophagy defects* | The ability of neurons to degrade proteins and organelles, through autophagy, is defective in neurons of AD patients |
| *Cholesterol metabolism* | High occurrence of adipose inclusions are observed in AD brains; APOE, a regulator of cholesterol metabolism, if the most significant risk factor for sporadic AD; high serum cholesterol is a risk factor for AD; cholesterol lowering drugs have been shown to be protective in AD prospective trials |

**Factors that play a role in the pathogenesis of Alzheimer's disease**. The interplay of how and when these processes come together to confer risk of disease remains unknown, in particular at the molecular level (Hardy and Selkoe, 2002; Mattson, 2004; Herrup, 2010; Querfurth and Laferla, 2010; Di Paolo and Kim, 2011).
