# Supplementary material for: Pathway-Based Analysis of Genome-Wide siRNA Screens Reveals the Regulatory Landscape of App Processing
Source: PLoS One. 2015 Feb 27;10(2):e0115369. doi: 10.1371/journal.pone.0115369 (PMC4344212; doi:10.1371/journal.pone.0115369)
Supplement: S10 Supplementary Information — (DOCX) [file pone.0115369.s010.docx]

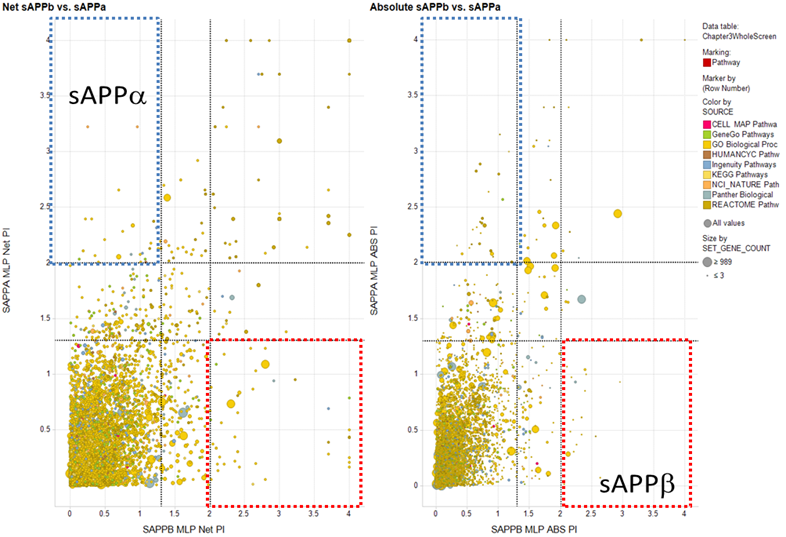


**Significant for sAPPα (p <0.01) but not sAPPβ (p > 0.05 )** : amyloid precursor protein catabolic and metabolic process(GO Biological Process), ATP/ITP metabolism(GeneGo Pathways), Dual incision reaction in TC-NER(REACTOME Pathways), Formation of transcription-coupled NER (TC-NER) repair complex(REACTOME Pathways), Maturation of Notch precursor via proteolytic cleavage(REACTOME Pathways), Mature Notch receptor traffics to plasma membrane(REACTOME Pathways), mRNA Splicing - Minor Pathway(REACTOME Pathways), negative regulation of transcription from RNA polymerase II promoter(GO Biological Process), Notch receptor binds with a ligand(REACTOME Pathways), Notch signaling pathway(KEGG Pathways), Receptor-ligand binding initiates the second proteolytic cleavage of Notch receptor(REACTOME Pathways), regulation of T-helper 2 cell differentiation(GO Biological Process), regulation of T-helper 2 type immune response(GO Biological Process), response to protein stimulus(GO Biological Process), RNA polymerase(KEGG Pathways), Signaling by BMP(REACTOME Pathways), Signaling by Notch(REACTOME Pathways), T-helper 2 cell differentiation(GO Biological Process), Transcription-coupled NER (TC-NER)(REACTOME Pathways), Transport of Notch receptor precursor to golgi(REACTOME Pathways), Viral Messenger RNA Synthesis(REACTOME Pathways)

**Significant fo sAPPβ (p <0.01) but not sAPPα (p > 0.05 ):** Adipocytokine signaling pathway(KEGG Pathways), anterior/posterior axis specification(GO Biological Process), axis specification(GO Biological Process), blastoderm segmentation(GO Biological Process), Caveolar-mediated Endocytosis (3j6wi)(Ingenuity Pathways), cell fate commitment(GO Biological Process), cellular macromolecule catabolic process(GO Biological Process), COPI coating of Golgi vesicle(GO Biological Process), COPI Mediated Transport(REACTOME Pathways), cytokine secretion during immune response(GO Biological Process), dATP/dITP metabolism(GeneGo Pathways), defense response(GO Biological Process), determination of anterior/posterior axis, embryo(GO Biological Process), development of primary male sexual characteristics(GO Biological Process), development of primary sexual characteristics(GO Biological Process), embryonic axis specification(GO Biological Process), embryonic pattern specification(GO Biological Process), endocrine pancreas development(GO Biological Process) ,Golgi to ER Retrograde Transport(REACTOME Pathways), Golgi transport vesicle coating(GO Biological Process), Golgi vesicle budding(GO Biological Process), intra-Golgi vesicle-mediated transport(GO Biological Process), male sex determination(GO Biological Process),male sex differentiation(GO Biological Process), Maturity onset diabetes of the young(KEGG Pathways), membrane budding(GO Biological Process), Membrane Trafficking(REACTOME Pathways), MyD88-dependent toll-like receptor signaling pathway(GO Biological Process), negative regulation of cytokine secretion during immune response(GO Biological Process), negative regulation of protein secretion(GO Biological Process), neuron fate commitment(GO Biological Process), oligodendrocyte development(GO Biological Process), oligodendrocyte differentiation(GO Biological Process), pancreas development(GO Biological Process), pancreatic juice secretion(GO Biological Process), Plug Formation(REACTOME Pathways), regulation of cytokine secretion during immune response(GO Biological Process), Regulation of gene expression in late stage (branching morphogenesis) pancreatic bud precursor cells(REACTOME Pathways), regulation of isotype switching(GO Biological Process), response to inorganic substance(GO Biological Process), response to osmotic stress(GO Biological Process), response to salt stress(GO Biological Process), retrograde vesicle-mediated transport, Golgi to ER(GO Biological Process), sex determination(GO Biological Process), Skeletal development(Panther Biological Process), superoxide release(GO Biological Process), Transport_RAB1A regulation pathway(GeneGo Pathways), tripartite regional subdivision(GO Biological Process), vesicle coating(GO Biological Process), vesicle localization(GO Biological Process), vesicle targeting(GO Biological Process), vesicle targeting, to, from or within Golgi(GO Biological Process)
