# Supplementary material for: Pathway-Based Analysis of Genome-Wide siRNA Screens Reveals the Regulatory Landscape of App Processing
Source: PLoS One. 2015 Feb 27;10(2):e0115369. doi: 10.1371/journal.pone.0115369 (PMC4344212; doi:10.1371/journal.pone.0115369)
Supplement: S11 Supplementary Information — (DOCX) [file pone.0115369.s011.docx]

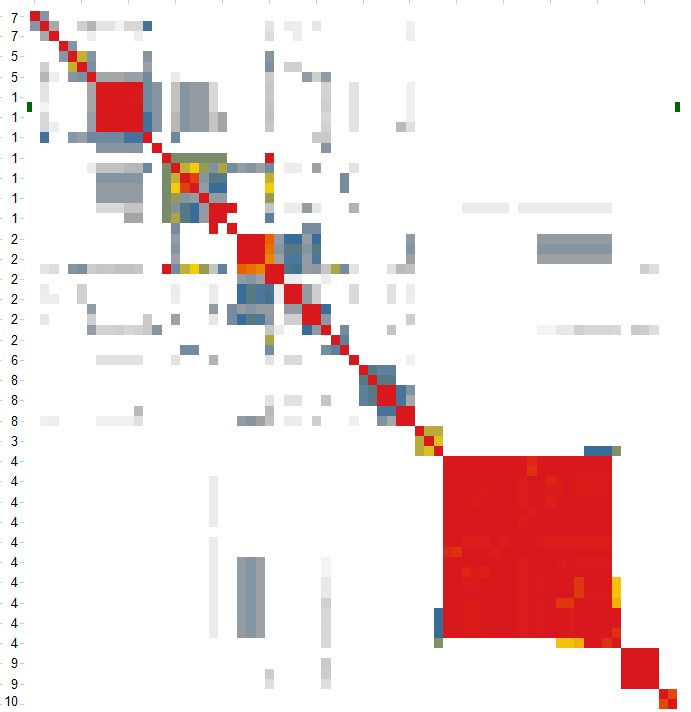


**Aβ40 Regulatory Landscape**

| **Cluster** | **Pathways/Processes** |
| --- | --- |
| 1 | Regulation of autophagy(GO Biological Process), Plug Formation(REACTOME Pathways), PECAM1 interactions(REACTOME Pathways), p130Cas linkage to MAPK signaling for integrins(REACTOME Pathways), neuron development(GO Biological Process), neurite development(GO Biological Process), memory(GO Biological Process), integrin-mediated signaling pathway(GO Biological Process), Grb2:SOS provides linkage to MAPK signaling for Intergrins(REACTOME Pathways), cell-substrate junction assembly(GO Biological Process), cell-cell junction maintenance(GO Biological Process), cell projection morphogenesis(GO Biological Process), cell morphogenesis involved in neuron differentiation(GO Biological Process), cell junction organization(GO Biological Process), axonogenesis(GO Biological Process) |
| 2 | response to mechanical stimulus(GO Biological Process), regulation of granulocyte macrophage colony-stimulating factor production(GO Biological Process), regulation of granulocyte macrophage colony-stimulating factor biosynthetic process(GO Biological Process), protein destabilization(GO Biological Process), positive regulation of granulocyte macrophage colony-stimulating factor biosynthetic process(GO Biological Process), nitric oxide metabolic process(GO Biological Process), nitric oxide biosynthetic process(GO Biological Process), negative regulation of defense response to virus(GO Biological Process), Natural killer cell mediated immunity(Panther Biological Process), mRNA transcription regulation(Panther Biological Process), mechanosensory behavior(GO Biological Process), Immunity and defense(Panther Biological Process) |
| 3 | Formation of incision complex in GG-NER(REACTOME Pathways), DNA replication(KEGG Pathways), Base Excision Repair(REACTOME Pathways) |
| 4 | Viral mRNA Translation(REACTOME Pathways), Transport of the SLBP independent Mature mRNA(REACTOME Pathways), Transport of Mature Transcript to Cytoplasm(REACTOME Pathways) Transport of Mature mRNAs Derived from Intronless Transcripts(REACTOME Pathways), RNA Pol II CTD phosphorylation and interaction with CE(REACTOME Pathways), Processing of Capped Intron-Containing Pre-mRNA(REACTOME Pathways), mRNA Splicing - Major Pathway(REACTOME Pathways), mRNA Processing(REACTOME Pathways), mRNA Capping(REACTOME Pathways), L13a-mediated translational silencing of Ceruloplasmin expression(REACTOME Pathways), GTP hydrolysis and joining of the 60S ribosomal subunit(REACTOME Pathways), Eukaryotic Translation Termination & Elongation(REACTOME Pathways), Elongation arrest and recovery(REACTOME Pathways), Elongation and Processing of Capped Transcripts(REACTOME Pathways), Cap-dependent Translation Initiation(REACTOME Pathways) |
| 5 | Other receptor mediated signaling pathway(Panther Biological Process), NRIF signals cell death from the nucleus(REACTOME Pathways), Alzheimer's disease(KEGG Pathways) |
| 6 | calcium-dependent cell-cell adhesion(GO Biological Process) |
| 7 | Synaptic transmission(Panther Biological Process), protein amino acid prenylation(GO Biological Process), detection of chemical stimulus(GO Biological Process) |
| 8 | transition metal ion transport(GO Biological Process), copper ion transport(GO Biological Process), cofactor catabolic process(GO Biological Process), cellular respiration(GO Biological Process), aerobic respiration(GO Biological Process), acetyl-CoA metabolic process(GO Biological Process) |
| 9 | N-acetylglucosamine metabolic process(GO Biological Process), glucosamine metabolic process(GO Biological Process), amino sugar metabolic process(GO Biological Process), amino sugar catabolic process(GO Biological Process) |
| 10 | Selenoamino acid metabolism(KEGG Pathways), peptidyl-arginine methylation(GO Biological Process) |


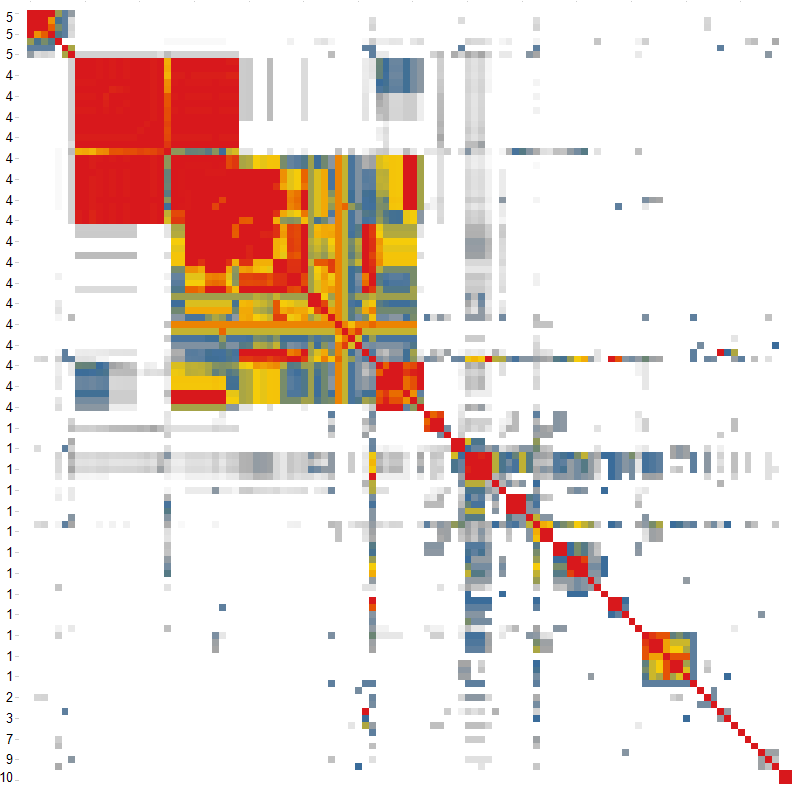
a

**sAPPα Regulatory Landscape**

| **Cluster** | **Pathways/Processes** |
| --- | --- |
| 1 | T-helper 2 cell differentiation(GO Biological Process), steroid hormone receptor signaling pathway(GO Biological Process), Signaling by BMP(REACTOME Pathways), response to protein stimulus(GO Biological Process), regulation of transcription in response to stress(GO Biological Process), regulation of transcription from RNA polymerase II promoter in response to oxidative stress(GO Biological Process), regulation of T-helper 2 type immune response(GO Biological Process), regulation of T-helper 2 cell differentiation(GO Biological Process), regulation of Rho GTPase activity(GO Biological Process), regulation of immunoglobulin mediated immune response(GO Biological Process), regulation of chondrocyte differentiation(GO Biological Process), regulation of B cell mediated immunity(GO Biological Process), Receptor-ligand binding initiates the second proteolytic cleavage of Notch receptor(REACTOME Pathways), Ras signaling in the CD4+ TCR pathway(NCI_NATURE Pathways), positive regulation of cell proliferation(GO Biological Process), Notch signaling pathway(KEGG Pathways), Notch receptor processing(GO Biological Process), negative regulation of transcription(GO Biological Process), negative regulation of transcription from RNA polymerase II promoter(GO Biological Process, negative regulatio,n of osteoclast differentiation(GO Biological Process), negative regulation of nucleobase, nucleoside, nucleotide and nucleic acid metabolic process(GO Biological Process), negative regulation of macromolecule biosynthetic process(GO Biological Process), negative regulation of interferon-gamma production(GO Biological Process), myeloid leukocyte differentiation(GO Biological Process), morphogenesis of a branching structure(GO Biological Process), membrane protein ectodomain proteolysis(GO Biological Process), Maturation of Notch precursor via proteolytic cleavage(REACTOME Pathways), hemopoietic progenitor cell differentiation(GO Biological Process), erythrocyte development(GO Biological Process), embryonic hemopoiesis(GO Biological Process), EGFR downregulation(REACTOME Pathways), deoxyribonucleoside metabolic process(GO Biological Process), cofactor catabolic process(GO Biological Process), chondrocyte differentiation(GO Biological Process), cartilage development(GO Biological Process), branching morphogenesis of a tube(GO Biological Process), amyloid precursor protein metabolic process(GO Biological Process), amyloid precursor protein catabolic process(GO Biological Process) |
| 2 | phosphatidic acid metabolic process(GO Biological Process), negative regulation of heart contraction(GO Biological Process), Atherosclerosis_Role of ZNF202 in regulation of expression of genes involved in Atherosclerosis(GeneGo Pathways) |
| 3 | Caspase cascade in apoptosis(NCI_NATURE Pathways), antigen processing and presentation of endogenous peptide antigen via MHC class I(GO Biological Process) |
| 4 | Viral Messenger RNA Synthesis(REACTOME Pathways), Transport of the SLBP independent Mature mRNA(REACTOME Pathways), Transport of the SLBP Dependant Mature mRNA(REACTOME Pathways), Transport of Mature Transcript to Cytoplasm(REACTOME Pathways), Transport of Mature mRNAs Derived from Intronless Transcripts(REACTOME Pathways), Transport of Mature mRNA Derived from an Intronless Transcript(REACTOME Pathways), Transport of Mature mRNA derived from an Intron-Containing Transcript(REACTOME Pathways), translation(GO Biological Process), Transcription-coupled NER (TC-NER)(REACTOME Pathways), Transcription(REACTOME Pathways), Transcription of the HIV genome(REACTOME Pathways), transcription from RNA polymerase II promoter(GO Biological Process), Tat-mediated HIV-1 elongation arrest and recovery(REACTOME Pathways), spliceosome assembly(GO Biological Process), RNA splicing, via transesterification reactions(GO Biological Process), RNA splicing, via transesterification reactions with bulged adenosine as nucleophile(GO Biological Process), RNA splicing(GO Biological Process), RNA polymerase(KEGG Pathways), RNA Polymerase II Transcription(REACTOME Pathways), RNA Pol II CTD phosphorylation and interaction with CE(REACTOME Pathways), RNA elongation(GO Biological Process), RNA elongation from RNA polymerase II promoter(GO Biological Process), Repair synthesis for gap-filling by DNA polymerase in TC-NER(REACTOME Pathways), Regulatory RNA pathways(REACTOME Pathways), Processing of Capped Intron-Containing Pre-mRNA(REACTOME Pathways), Pausing and recovery of HIV-1 elongation(REACTOME Pathways), Nucleotide Excision Repair Pathway (ci3)(Ingenuity Pathways), mRNA Splicing - Minor Pathway(REACTOME Pathways), mRNA Splicing - Major Pathway(REACTOME Pathways), mRNA Processing(REACTOME Pathways), mRNA processing(GO Biological Process), mRNA Editing(REACTOME Pathways), mRNA Capping(REACTOME Pathways), mRNA 3'-end processing(REACTOME Pathways), L13a-mediated translational silencing of Ceruloplasmin expression(REACTOME Pathways), HIV-1 Transcription Initiation(REACTOME Pathways), HIV-1 Transcription Elongation(REACTOME Pathways), HIV Infection(REACTOME Pathways), GTP hydrolysis and joining of the 60S ribosomal subunit(REACTOME Pathways), General mRNA transcription activities(Panther Biological Process), Gene Expression(REACTOME Pathways), Formation of the ternary complex, and subsequently, the 43S complex(REACTOME Pathways), Formation of the Editosome(REACTOME Pathways), Formation of RNA Pol II elongation complex(REACTOME Pathways), Formation of HIV-1 elongation complex containing HIV-1 Tat(REACTOME Pathways), Estrogen Receptor Signaling (chy)(Ingenuity Pathways), Elongation arrest and recovery(REACTOME Pathways), Elongation and Processing of Capped Transcripts(REACTOME Pathways), Cap-dependent Translation Initiation(REACTOME Pathways), ATP/ITP metabolism(GeneGo Pathways), Abortive elongation of HIV-1 transcript in the absence of Tat(REACTOME Pathways) |
| 5 | vitamin A metabolic process(GO Biological Process), visual perception(GO Biological Process), retinol metabolic process(GO Biological Process), retinoid metabolic process(GO Biological Process), positive regulation of insulin secretion(GO Biological Process), glucose homeostasis(GO Biological Process), fat-soluble vitamin metabolic process(GO Biological Process) |
| 6 | Endoderm development(Panther Biological Process) |
| 7 | Glycosphingolipid Biosynthesis - Neolactoseries (1blge)(Ingenuity Pathways) |
| 8 | protein targeting to Golgi(GO Biological Process) |
| 9 | superpathway of glycolysis, pyruvate dehydrogenase, TCA, and glyoxylate bypass(HUMANCYC Pathways), GTP metabolism(GeneGo Pathways), Glycolysis and gluconeogenesis (short map)(GeneGo Pathways) |
| 10 | unsaturated fatty acid metabolic and biosynthetic process(GO Biological Process) |


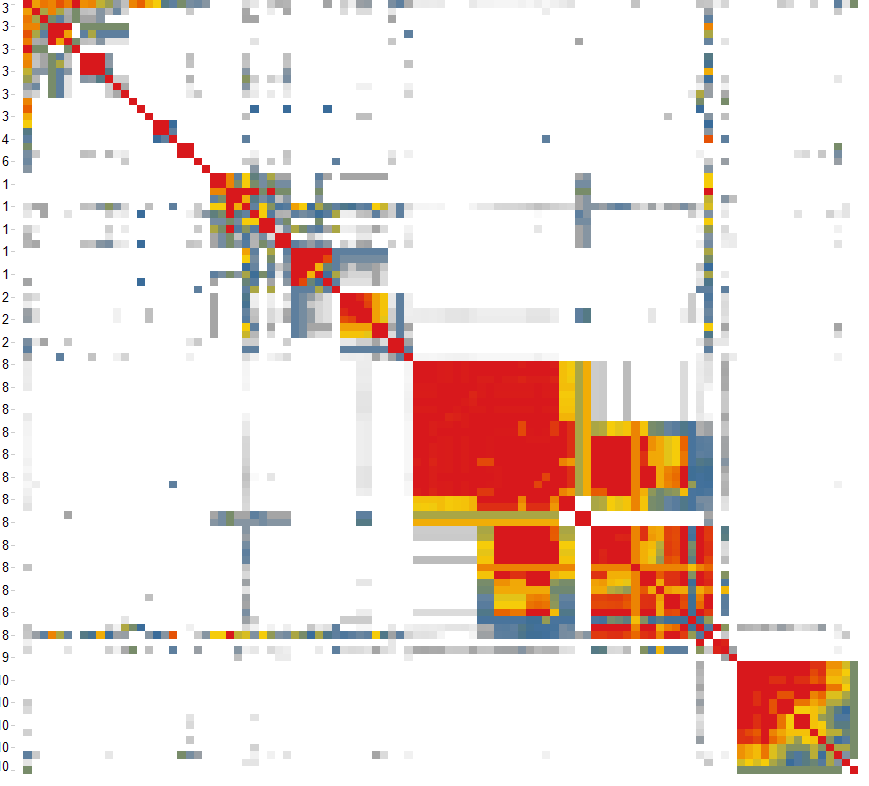


**sAPPβ Regulatory Landscape**

| **Cluster** | **Pathways/Processes** |
| --- | --- |
| 1 | pancreas development(GO Biological Process), endocrine pancreas development(GO Biological Process), Regulation of gene expression in late stage (branching morphogenesis) pancreatic bud precursor cells(REACTOME Pathways), Maturity onset diabetes of the young(KEGG Pathways), mRNA transcription regulation(Panther Biological Process), brain development(GO Biological Process), neuron fate commitment(GO Biological Process), cell fate commitment(GO Biological Process), Skeletal development(Panther Biological Process), Mesoderm development(Panther Biological Process), determination of anterior/posterior axis, embryo(GO Biological Process), anterior/posterior axis specification(GO Biological Process), axis specification(GO Biological Process), anterior/posterior pattern formation(GO Biological Process), embryonic pattern specification(GO Biological Process), hemopoietic progenitor cell differentiation(GO Biological Process) |
| 2 | male sex differentiation(GO Biological Process), development of primary male sexual characteristics(GO Biological Process), development of primary sexual characteristics(GO Biological Process), gonad development(GO Biological Process), male sex determination(GO Biological Process), sex determination(GO Biological Process), response to osmotic stress(GO Biological Process), response to salt stress(GO Biological Process), response to inorganic substance(GO Biological Process) |
| 3 | defense response(GO Biological Process), TREM1 Signaling (3j6wq)(Ingenuity Pathways), type I interferon production(GO Biological Process), negative regulation of cytokine secretion during immune response(GO Biological Process), cytokine secretion during immune response(GO Biological Process), negative regulation of protein secretion(GO Biological Process), MyD88-dependent toll-like receptor signaling pathway(GO Biological Process), regulation of immunoglobulin mediated immune response(GO Biological Process), regulation of B cell mediated immunity(GO Biological Process), regulation of isotype switching(GO Biological Process), Calcineurin-regulated NFAT-dependent transcription in lymphocytes(NCI_NATURE Pathways), Adipocytokine signaling pathway(KEGG Pathways), Caspase cascade in apoptosis(NCI_NATURE Pathways), DNA deamination(GO Biological Process), positive regulation of leukocyte migration(GO Biological Process), superoxide release(GO Biological Process) |
| 4 | porphyrin catabolic process(GO Biological Process), cofactor catabolic process(GO Biological Process), regulation of transcription in response to stress(GO Biological Process) |
| 5 | Plug Formation(REACTOME Pathways), Blood coagulation_GPIb-IX-V-dependent platelet activation(GeneGo Pathways) |
| 6 | memory(GO Biological Process) |
| 7 | gamma-aminobutyric acid transport(GO Biological Process) |
| 8 | Formation of the Editosome(REACTOME Pathways), mRNA 3'-end processing(REACTOME Pathways), mRNA Editing(REACTOME Pathways), Transport of Mature Transcript to Cytoplasm(REACTOME Pathways), Transport of Mature mRNAs Derived from Intronless Transcripts(REACTOME Pathways), Transport of Mature mRNA Derived from an Intronless Transcript(REACTOME Pathways), Transport of the SLBP Dependant Mature mRNA(REACTOME Pathways), Transport of Mature mRNA derived from an Intron-Containing Transcript(REACTOME Pathways), mRNA Splicing - Major Pathway(REACTOME Pathways), Processing of Capped Intron-Containing Pre-mRNA(REACTOME Pathways), mRNA Processing(REACTOME Pathways), mRNA Capping(REACTOME Pathways), Elongation and Processing of Capped Transcripts(REACTOME Pathways), Formation of RNA Pol II elongation complex(REACTOME Pathways), RNA Polymerase II Transcription(REACTOME Pathways), Gene Expression(REACTOME Pathways), Transcription(REACTOME Pathways), Elongation arrest and recovery(REACTOME Pathways), Influenza Viral RNA Transcription and Replication(REACTOME Pathways), Influenza Infection(REACTOME Pathways), oligodendrocyte differentiation(GO Biological Process), oligodendrocyte development(GO Biological Process), HIV-1 Transcription Elongation(REACTOME Pathways), Formation of HIV-1 elongation complex containing HIV-1 Tat(REACTOME Pathways), Tat-mediated HIV-1 elongation arrest and recovery(REACTOME Pathways), Pausing and recovery of HIV-1 elongation(REACTOME Pathways), Abortive elongation of HIV-1 transcript in the absence of Tat(REACTOME Pathways), Regulatory RNA pathways(REACTOME Pathways), RNA Pol II CTD phosphorylation and interaction with CE(REACTOME Pathways), HIV-1 Transcription Initiation(REACTOME Pathways), Nucleotide Excision Repair Pathway (ci3)(Ingenuity Pathways), RNA elongation(GO Biological Process), RNA elongation from RNA polymerase II promoter(GO Biological Process), Transcription of the HIV genome(REACTOME Pathways), General mRNA transcription activities(Panther Biological Process), HIV Infection(REACTOME Pathways), transcription from RNA polymerase II promoter(GO Biological Process), cellular macromolecule catabolic process(GO Biological Process) |
| 9 | dATP/dITP metabolism(GeneGo Pathways) |
| 10 | COPI coating of Golgi vesicle(GO Biological Process), Golgi transport vesicle coating(GO Biological Process), Golgi vesicle budding(GO Biological Process), Golgi to ER Retrograde Transport(REACTOME Pathways), vesicle targeting, to, from or within Golgi(GO Biological Process), vesicle coating(GO Biological Process), membrane budding(GO Biological Process), vesicle localization(GO Biological Process), vesicle targeting(GO Biological Process), retrograde vesicle-mediated transport, Golgi to ER(GO Biological Process), Membrane Trafficking(REACTOME Pathways), Transport_RAB1A regulation pathway(GeneGo Pathways), Caveolar-mediated Endocytosis (3j6wi)(Ingenuity Pathways), intra-Golgi vesicle-mediated transport(GO Biological Process), pancreatic juice secretion(GO Biological Process) |
